# How subduction evolution drives sediment-hosted mineralisation along craton edges

**DOI:** 10.1038/s41467-026-74134-5

**Published:** 2026-06-10

**Authors:** Hojat Shirmard, Ben Mather, Ehsan Farahbakhsh, Craig O’Neill, R. Dietmar Müller

**Affiliations:** 1 EarthByte Group, School of Geosciences, The University of Sydney, Sydney, Australia; 2 School of Geography, Earth and Atmospheric Sciences, Faculty of Science, The University of Melbourne, Melbourne, Australia; 3 School of Earth and Atmospheric Science, Queensland University of Technology, Brisbane, Australia

**Keywords:** Tectonics, Geology

## Abstract

Sediment-hosted mineral deposits cluster near craton edges, yet the geodynamic factors influencing this concentration remain poorly understood. To investigate the tectonic and geodynamic controls on craton-edge mineralisation, we integrate a 1.8 Ga global plate motion model with craton-edge mapping from full-waveform seismic tomography, geodynamic modelling, and a global mineral deposit database. Here we show that mineralised craton edges consistently cluster 800–1800 km from subduction zones at the time of formation, with some as far away as ~3000 km—a spatial pattern distinct from random craton-edge locations. Geodynamic models show that subduction generates broad mantle return-flow cells that focus lithospheric stress, strain, and weakening at comparable distances from the trench. We propose that this mechanically driven weakening promotes rifting, enhances permeability, and facilitates the infiltration of slab-derived volatiles, thereby preconditioning the lithosphere for metallogenesis. These results identify subduction–craton coupling as a first-order control on sediment-hosted mineral systems across multiple supercontinent cycles and provide a predictive geodynamic framework for understanding the distribution of continental resources through deep time.Subduction near cratons fosters conditions that enrich the mantle and promote mineral formation along craton edges.

## Abstract (web summary)

This study shows that subduction near cratons fosters conditions that enrich the mantle and promote mineral formation along craton edges.

## Introduction

Craton edges, the enduring fault zones of continents, represent tectonic seams where mantle flow, lithospheric weakening, and metal enrichment converge, shaping Earth’s mineral wealth over geological time^1,2^. Craton edges have long been recognised as favourable loci for metal accumulation because they host faults, shear zones, and reactivated structures that facilitate large-scale hydrothermal circulation^3–5^. These fluid pathways, when intersecting redox fronts or reactive host rocks, facilitate the precipitation of metals such as zinc (Zn), lead (Pb), and copper (Cu), contributing to sediment-hosted deposit formation^6–8^. Many of the world’s largest clastic-dominated (CD), Mississippi Valley-type (MVT), and sediment-hosted copper (Cu-Sed) deposits occur in rifts associated with cratons, including gold (Au), Cu, and rare earth elements, underlain by a mantle lithosphere enriched in volatiles like carbon, sulfur, and water—crucial elements for metallogenesis^6,9^. Despite decades of study linking these deposits to rifting, basinal brines, and structural reactivation^10,11^, a fundamental question remains unresolved: why are only certain craton edges repeatedly mineralised, whereas others with similar stratigraphy and structures remain barren?

Traditional genetic models emphasise processes operating within sedimentary basins—metal sourcing, fluid circulation, and chemical trapping—but these mechanisms do not explain the large-scale spatial predictability of mineralised craton edges through time. The formation of sediment-hosted craton-edge deposits requires a combination of metal source, transport, and trap. Basin-scale brine circulation mobilises metals from source rocks, often aided by sequential faulting and asymmetric rifting that focuses permeability and heat flow^10,11^. Marine mudstones then provide the necessary chemical gradients for precipitation, typically over short (1–3 Myr) mineralisation windows near paleo-shorelines^12^. While such basin-scale models are well supported, they do not explain why some craton edges are metal-rich, whereas others are not.

Recent advances in full-waveform seismic tomography^13^ and machine learning–based craton boundary mapping reveal that over 80% of craton-related sediment-hosted deposits, including all giant deposits, are located within ~90 km of the present-day craton edges^14^. This highlights the importance of precisely defining craton edges, but the geodynamic processes that make some boundaries fertile remain poorly understood. Most studies have treated sediment-hosted deposits as rift and passive-edge phenomena^15–17^, disconnected from magmatism and particularly from subduction.

Subduction, however, serves as a primary driver of plate motions and mantle flow. Deep, long-lived subduction can induce trench rollback, extensional stresses, and back-arc rifting thousands of kilometres from the trench^18,19^. This raises the question: do subduction systems control the timing and spatial distribution of mineralisation along craton edges by influencing lithospheric extension and volatile budgets?

Here, we evaluate the hypothesis that subduction–craton coupling governs the formation of sediment-hosted mineral systems in space and time. We integrate a global plate motion model spanning 1.8 Ga with craton-edge mapping, age-coded deposit data, and numerical geodynamic simulations to address this question. We quantify the spatiotemporal relationship between craton edges and subduction zones, compare observed deposit distributions with those of random points near craton edges, and test geodynamic scenarios that vary trench–craton distances. By linking mineral system formation to plate boundary evolution, we provide a predictive framework that connects craton-edge metallogenesis to supercontinent cycles and mantle dynamics.

## Results

### Global spatiotemporal patterns

**Fig. 1:**
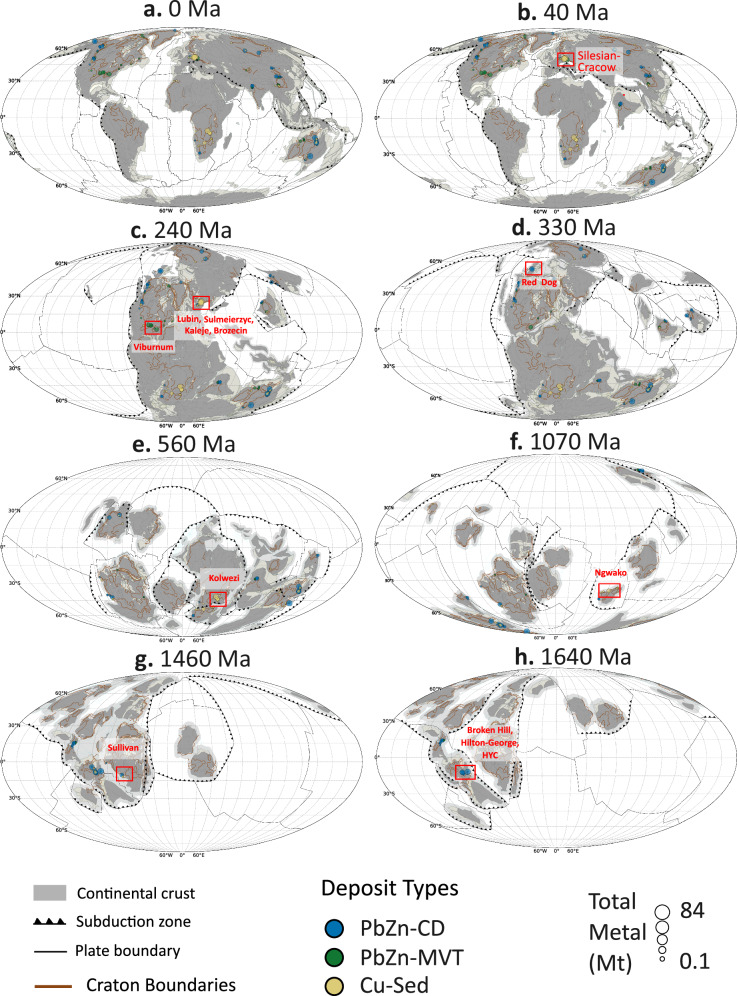
Temporal distribution of major mineral deposits, craton boundaries, continents, and trench lines. Mineral deposits are shown in relation to subduction. Deposit size is shown in megatonnes (Mt) and increases from 0.1 to 84 Mt. **a** 0 Ma (present day), **b** 40 Ma (the Silesian–Cracow mineral district), **c** 240 Ma (the Viburnum and the Lublin–Sulmierzyce–Kaleje–Brozenin mineral district), **d** 330 Ma (the Red Dog deposit), **e** 560 Ma (the Kolwezi deposit), **f** 1070 Ma (the Ngwako deposit), **g** 1460 Ma (the Sullivan deposit), **h** 1640 Ma (the Broken Hill, Hilton–George, and HYC deposits).

To accurately place the plate reconstructions within a geodynamically self-consistent mantle reference frame, we adopt an absolute plate motion model derived using an optimisation technique that enforces empirical geodynamic constraints known to govern plate motion relative to the mantle^20^. This overcomes the limitations of absolute plate motion models based on paleomagnetic data, including their inability to constrain longitudinal plate motions and their tendency to introduce unrealistic net lithospheric rotation and subduction zone migration^21^. The spatial relationship between mineral deposits and major supercontinental cycles highlights how tectonic configurations during assembly and breakup phases may have influenced ore-forming processes. The reconstruction provides a plate tectonic context for interpreting mineralisation patterns through time (Fig. 1a–h), (Supplementary Materials Movie S1).

**Fig. 2:**
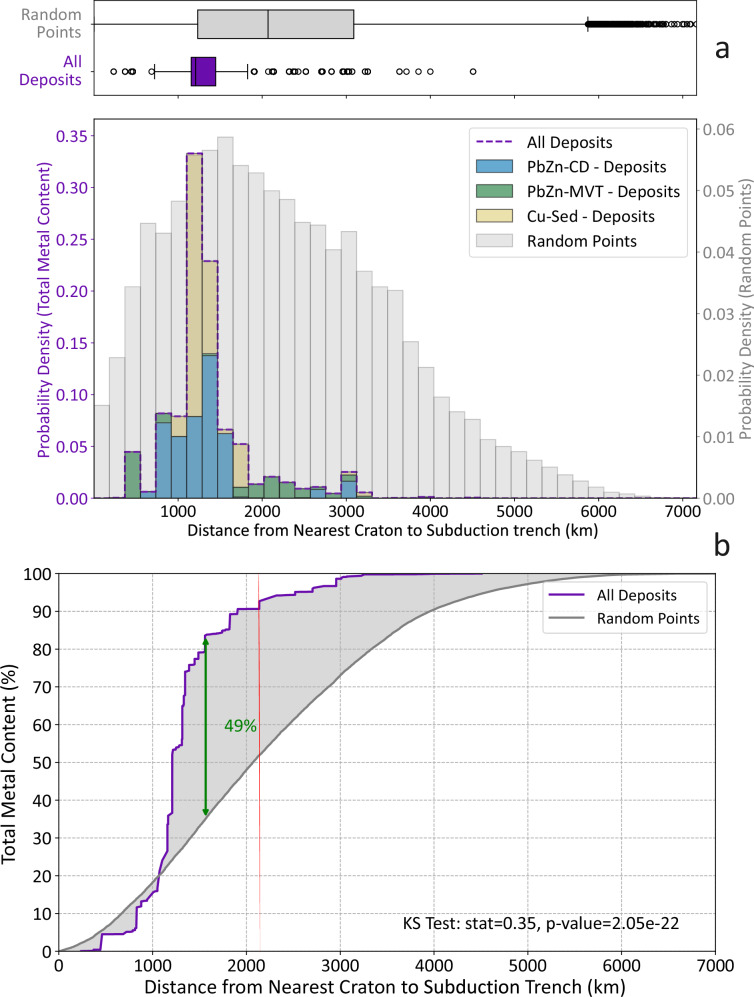
Sediment-hosted metal deposits cluster near ancient subduction zones. **a** Distribution of distances between craton edges and the nearest subduction trench for sediment-hosted deposits (purple line) and 90,000 random craton-edge points (grey). Deposits cluster within 800–1800 km of trenches, with a median of 1200 km, nearly 900 km shorter than random points. **b** Cumulative distribution of total contained metal versus trench distance. Over 90% of the metal content lies within 2200 km of the trenches (red line). The Kolmogorov–Smirnov statistic (D = 0.35, *p* ≪ 0.001) confirms that deposits are significantly biased toward subduction-proximal craton edges.

We quantified the distance between craton edges and subduction trenches from 1.8 Ga to the present, comparing the locations of 2166 age-coded mineral deposits with 90,000 random points generated within a 185 km buffer zone around craton edges. Mineralised craton edges are located systematically within 800–1800 km of trenches at the time of deposit formation, with some as far away as ~3000 km (Fig. 2a). In contrast, random points are more broadly distributed, with distances extending up to 7000 km. Median trench distance for deposits is 1200 km—nearly 900 km shorter than the 2070 km median of random points. We also observe negative trench-normal motion values (see Supplementary Materials, Fig. S1), indicating trench retreat, where the overriding plate migrates oceanward and repositions cratons over previously subducted domains.

Cumulative distribution analysis underscores the significance of this spatial bias. Over 90% of the total metal content is located within 2200 km of the nearest trench (Fig. 2b). The Kolmogorov–Smirnov (K–S) statistic^22^ (D = 0.35, *p* = 2.0 × 10^−22^) confirms that the distributions of mineral deposits and random points are significantly different. The K–S statistic *D* represents the maximum vertical separation between the cumulative distribution functions of the two samples; higher values indicate greater divergence between distributions, whereas lower values indicate increasing similarity (e.g., Massey, ^22^). This result indicates that proximity to subduction zones is a robust discriminator between fertile and barren craton edges.

**Fig. 3:**
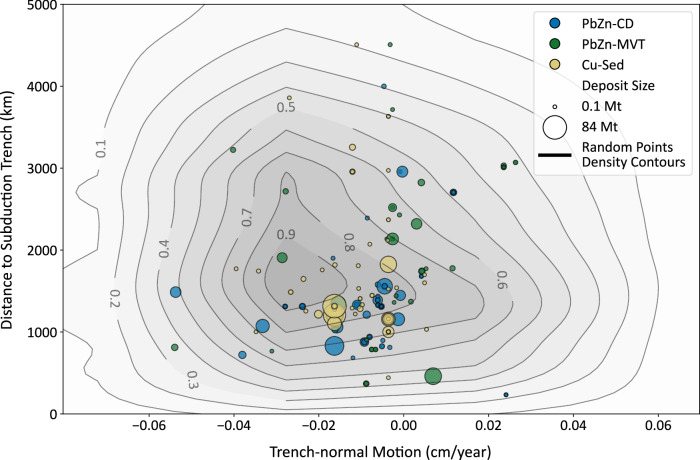
Preference of sediment-hosted deposits for moderate trench retreat. Trench-normal motion versus distance to the subduction trench for giant Cu-Sed, Pb–Zn MVT, and Pb–Zn CD deposits, alongside a kernel density distribution of random points near the craton edges. Negative trench-normal motion corresponds to trench retreat, and positive values indicate trench advance. Deposit sizes are scaled by metal endowment. The grey contour lines represent the probability density of random points, with the highest-density region extending into both the negative and positive trench-normal motion zones. However, deposits are predominantly located in the negative trench-normal motion zone. Their trench-normal motion values are generally less negative than those associated with the most extreme trench retreat. Density is dimensionless because it represents a probability distribution scaled to a maximum of 1 in the highest-density area.

To place this distance window in a dynamic context, we analysed trench-normal plate motions. Many deposits formed during intervals of negative trench-normal motion, corresponding to trench retreat and oceanward migration of the subduction hinge (Fig. 3). This movement can result in cratons being positioned over enriched mantle domains, potentially fostering the formation of large Cu-Sed and Pb–Zn deposits. Although random craton-edge points occur across both retreat and advance regimes, deposits are preferentially associated with moderate trench retreat (Fig. 3), consistent with the inward transmission of deformation into continental interiors. This suggests that metallogenesis is favoured where subduction-driven extension is transmitted efficiently into continental interiors, but without wholesale lithospheric disruption.

### Geodynamic model

**Fig. 4:**
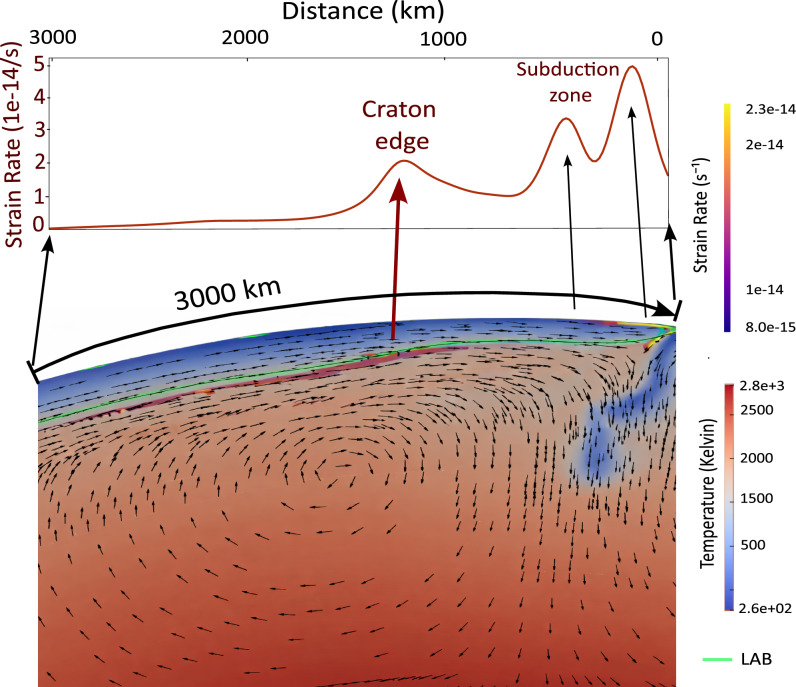
Subduction-driven mantle flow and strain localisation beneath craton edges. Numerical model of subduction-driven mantle return flow with a high peak at trench–craton distance of ~1300 km. Equi-length flow arrows show a broad subduction-related flow cell extending ~3000 km from the trench, with vertical flow velocities focused near the craton edge. Peak subcontinental strain rates occur at the craton edge, illustrating how subduction localises deformation far into the overriding plate. The strain rate colours can be seen near the lithosphere–asthenosphere boundary (LAB), indicated by the green line.

To assess the geodynamic controls on stress and strain focusing along craton edges, we use the ASPECT (Advanced Solver for Problems in Earth’s Convection) code^23^ for running 2D geodynamic models (see “Methods” for details). We ran an ensemble of 2D geodynamic models with trench–craton distances ranging from ~600 km to ~3400 km to test the physical plausibility of the 1000–2000 km distance window identified from the 1.8 Ga tectonic reconstruction (see “Methods” and Supplementary Materials). All models generate a broad mantle return flow cell that extends ~4000 km from the trench, focusing strain and stress at the craton edge (Fig. 4**)**. Strain-rate maxima occur when the distance from subduction is 1300 km, regardless of initial geometry (Fig. in Supplementary materials). These results demonstrate that subduction-driven mantle flow can transmit stresses thousands of kilometres into the overriding plate, promoting lithospheric weakening preferentially at craton edges. The observed clustering of deposits at distances of 800–1800 km overlaps with the modelled strain peaks but shows a slight offset. This likely reflects the natural complexity of three-dimensional mantle flow and the influence of inherited lithospheric heterogeneities, which can focus deformation more efficiently than represented in our simplified models.

Together, these results define a preferred window of trench–craton distance where lithospheric strain and mineralisation coincide. This window is globally consistent across supercontinent cycles and deposit types (Pb–Zn CD, Pb–Zn MVT, Cu–Sed). The spatial convergence between observed deposits and modelled deformation zones provides a quantitative link between subduction dynamics and the metallogenic fertility of craton edges.

In Fig. 4, an example of a numerical geodynamic model shows subduction-driven mantle return flow with a high peak at trench–craton distance of ~1300 km. The subduction return flow occurs at a scale similar to the craton edge distance, and its focusing in the sublithospheric mantle generates elevated strain rates beneath craton edges. The model shows a broad return flow cell extending nearly 3000 km from the trench, with strain-rate peaks at the craton edge.

**Fig. 5:**
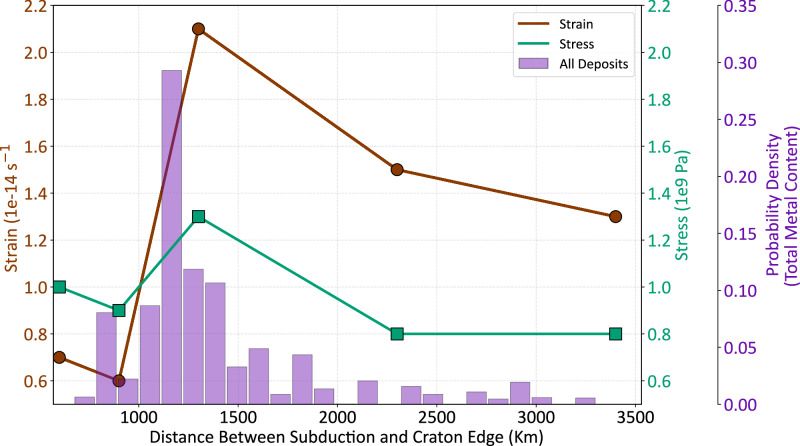
Peak lithospheric stress, strain, and the distribution of sediment-hosted deposits. Profiles of peak lithospheric strain rate (red) and differential stress (orange) from five models (see “Methods” for model parameters) with trench–craton distances between ~600 and 3400 km. The peak stress and strain rates resulting from the focusing of mantle return flow correlate well with the observed distance window for all targeted sediment-hosted deposits.

To further test the robustness of our results, we analysed the five numerical geodynamic models with varying initial distances between the subduction trench and craton edge. The highest strain rate levels occur when the craton edge is at around 1300 km distance from the subduction, approximately the foci of subduction-driven return flow (Fig. 5). This distance closely matches the reconstructed median distance from trenches to mineral deposits (~1200 km), linking the statistical signal to a physically generated deformation field. The effect is also present for differential stress (represented by the second strain rate invariant), but is weaker.

## Discussion

### Subduction zones and craton edge metallogenesis

Our results demonstrate that mineralisation along craton edges is systematically controlled by their distance from subduction zones. Globally, Pb–Zn clastic-dominated (CD), Mississippi Valley-type (MVT), and sediment-hosted copper (Cu-Sed) deposits cluster within 800–1800 km of contemporaneous trenches—a spatial window absent from points randomly generated along craton edges. This pattern is consistent across supercontinent cycles during assembly or break-up, suggesting that subduction dynamics exert a first-order influence on craton-edge metallogenesis.

Additionally, subduction rates have remained relatively stable, averaging around 5–7 cm/year since 1.8 Ga. The lengths of individual trenches and the slab geometry fluctuate over time, and we recognise that these changes may lead to local deviations from the global distance pattern. Our framework provides a broad, global-scale tectonic and geodynamic context, while acknowledging that variability in subduction styles can influence, shift, or limit the expression of this distance relationship in specific regions. This is why we observe that distances are not fixed but tend to cluster between 800 and 1800 km.

The preferred 800–1800 km distance range, therefore, represents a statistical window emerging from global data, not a fixed universal value for individual margins. Our numerical experiments show that, across a range of trench–craton configurations, subduction consistently generates broad return-flow cells with strain-rate maxima at ~1300 km from the trench. This suggests that, despite variations in slab geometry and convergence rate, the underlying scale of subduction-driven return flow provides a robust first-order control on where deformation—and hence potential metallogenic preconditioning—is most efficiently focused.

### Subduction-driven mantle flow and craton edge deformation

Numerical geodynamic models reproduce this preferred distance window, consistently showing strain and stress maxima 1000–2000 km from trenches. These results imply that subduction-driven mantle return flow localises deformation at craton edges, progressively weakening the lithosphere and reactivating inherited structures. By concentrating strain in surrounding mobile belts, this return flow preserves the long-term stability of cratons^24^, and promotes rifting and mineralisation along their edges^25^. This lithospheric weakening provides favourable conditions for the development of extensional basins along craton edges, where rift style, fault architecture, and basin evolution can strongly influence the efficiency and preservation of sediment-hosted mineral systems^26^.

Strain rates are vertically integrated between 100–300 km to isolate deformation within the mid- to lower lithosphere and the immediately underlying rheological boundary layer, where subduction-driven mantle return flow most effectively shears the base of the continental lithosphere. The upper bound of 300 km corresponds to the maximum cratonic thickness in the model and includes the basal portion of the cratonic root, which is commonly somewhat thinned in dynamic settings. The lower bound of 100 km approximates the initial thickness of the adjacent thinner continental lithosphere. This depth window avoids stress amplification in the shallow, high-viscosity lithosphere—where brittle processes are not explicitly resolved in our rheology—as well as the higher background strain rates of the deeper asthenosphere that are not directly related to lithosphere–mantle coupling. Although elevated strain is distributed more broadly beneath the continental plate, integration over this interval preferentially highlights strain localisation at craton edges. While the selection is not unique, it represents a physically motivated and internally consistent criterion that captures the strain features most relevant to lithospheric weakening at craton edges.

Our numerical models do not explicitly resolve continental deformation, fluid flow, or ore-forming processes. Instead, they isolate a first-order lithospheric-scale forcing mechanism that operates even within stable continental terranes. The models predict enhanced strain in the lower lithosphere and underlying mantle associated with subduction-driven return flow. We interpret this deep-seated strain as being mechanically coupled, over geological timescales, to smaller-scale and sub-resolution deformation within the continental lithosphere and upper crust, particularly along inherited craton edge structures. These conditions are widely recognised as prerequisites for basin development and fluid circulation in sediment-hosted mineral systems. Accordingly, our models provide a geodynamic framework that complements, rather than directly simulates, crustal deformation and metallogenesis.

The natural scale of this return flow means strain focusing on craton edges is particularly effective for subduction zones between 1000–2000 km from craton boundaries. Such deformation enhances permeability, allowing slab-derived volatiles to infiltrate the subcontinental lithospheric mantle (SCLM), metasomatize its mineralogy, and enrich it in key ligands such as halogens, carbon, and sulfur. These processes precondition craton edges for ore formation over geologic timescales.

The highest strain rate occurs at 1300 km, and deposits form at a median of 1200 km, indicating only a 100 km difference. The slight offset between observed deposit distances and modelled strain maxima likely reflects simplifications in model rheology, viscosity structure, and 2D flow geometry. Natural systems include inherited lithospheric heterogeneities and 3D mantle flow that may more efficiently and more closely focus deformation toward the trench. Nonetheless, the convergence of plate tectonic results and geodynamic model predictions provides strong evidence for a causal link between subduction dynamics and craton edge mineralisation.

We impose plate velocities here to both control the experimental configuration and explore sublithospheric flow under realistic plate motion. However, this configuration limits our ability to fully explore slab rollback, break-off, and other three-dimensional effects, including the three-dimensional architecture of cratonic roots. As a result, we focus on the asthenospheric return flow around craton edges in response to subduction, to which these models are well suited.

Although our models do not simulate rifting, permeability evolution, or fluid–rock interaction, they establish a first-order mechanical link between subduction-driven return flow and lithospheric weakening at craton edges. Within this framework, the promotion of rifting, enhancement of permeability, and facilitation of slab-derived volatile infiltration are inferred consequences of sustained strain localisation acting over geological timescales, consistent with independent geological and geochemical evidence for basin development and metasomatic enrichment along craton margins.

### Trench retreat, mantle enrichment, and temporal clustering of deposits

This mechanism aligns with independent geochemical evidence for metasomatic enrichment of the subcontinental lithospheric mantle (SCLM) by slab-derived fluids^27^, which supply volatiles and ligands essential for metal transport^28,29^. Episodes of moderate trench retreat likely position cratons over enriched mantle domains, allowing metal-bearing fluids and partial melts to ascend and precipitate ore systems in back-arc and interior settings (Fig. 3). Also, there is some overlap between deposit locations and randomly distributed points, indicating that this relationship is not strictly exclusive but instead shows a degree of spatial scatter.

Many giant Cu-Sed and Pb–Zn deposits coincide with episodes of trench retreat, which drive oceanward migration of the overriding plate and position cratons over previously enriched mantle domains. This configuration promotes the ascent of partial melts and metal-bearing fluids into sedimentary basins, triggering short-lived (1–3 Myr) mineralisation events. Our results provide a physical explanation for the observed temporal clustering of deposits near supercontinent breakup phases, when subduction rollback and back-arc extension were widespread. We interpret the localised lithospheric strain observed in geodynamic models as a deep-seated dynamic forcing mechanism that promotes (1) reactivation of inherited structures, (2) lithospheric thinning and extension, and (3) basin formation and fault permeability.

### The role of slab fluids and ligands in metallogenic fertility

Beyond metal availability, the presence of key ligands (e.g., chloride, bicarbonate, and reduced sulfur) is critical for metal transport^30,31^. Subduction-derived fluids are especially rich in these components and ligands from previously enriched mantle domains can infiltrate and alter the lithosphere over long timescales. This metasomatic alteration enhances the capacity of basinal brines to dissolve, transport, and deposit metals such as Zn, Pb, and Cu^32,33^. These processes are crucial for forming CD and MVT deposits under the right redox and thermal conditions^12^. Thus, the overlooked role of slab fluids in enriching craton-edge lithosphere may be a key determinant of metallogenic fertility.

### Plate-mantle interactions: insights into sediment-hosted mineral exploration

By quantifying the distance window linking subduction to craton edge mineralisation, our study not only provides a predictive tool for exploration but also illuminates a fundamental aspect of Earth system evolution. Subduction-driven mantle return flow emerges as a primary mechanism coupling plate boundary dynamics with lithospheric weakening and volatile cycling. This feedback may have influenced the thermal state of the lithosphere, crustal growth, and the release of volatiles to Earth’s surface through deep time. Consequently, the metallogenic record becomes a proxy for large-scale mantle flow patterns and supercontinent cycles, offering a way to interrogate the history of plate–mantle interactions and planetary cooling. Future work integrating geodynamic simulations with geochemical tracers (e.g., halogens, sulfur isotopes, noble gases) and global reconstructions will refine our understanding of how subduction orchestrates lithospheric fertility, surface environments, and the distribution of critical resources through Earth history. The framework developed here can also serve as a foundation for future predictive mapping.

### Uncertainties, limitations, future work

While our framework links subduction, mantle return flow, and craton-edge mineralisation at a global, first-order level, significant uncertainties remain at the scale of individual systems. Three-dimensional slab geometry, time-dependent rollback, and local lithospheric heterogeneity can shift or suppress strain localisation relative to the idealised distance window used here. Consequently, our results should be viewed as defining a tectonic envelope that enhances the probability of fertile craton edges, rather than a deterministic predictor for every present-day subduction margin.

## Methods

To test whether subduction systematically controls the spatial distribution of sediment-hosted mineral systems, we developed a workflow that integrates global plate reconstructions, machine learning-derived craton boundary maps, a time-resolved mineral deposit database, and numerical geodynamic simulations. Our workflow quantifies the spatiotemporal relationship between craton edges and subduction trenches over the past 1.8 billion years and evaluates whether the observed distance distribution of deposits to the trench can be explained by subduction-driven mantle flow. By combining statistical comparison against randomly sampled craton edges with physically based mantle convection models, we isolate the effect of trench–craton distance as an independent geodynamic variable controlling lithospheric strain and metallogenic potential.

### Plate tectonic model

We used a global plate motion model spanning 1.8 Ga to the present^34^, integrating published reconstructions of Nuna/Columbia, Rodinia, and Gondwana/Pangaea^21^. The model was implemented in GPlates^35^ and converted to a geodynamically self-consistent mantle reference frame by applying an absolute plate motion optimisation that minimises net lithospheric rotation and enforces trench migration constraints. This approach is more suitable for analysing plate-mantle interaction, as paleomagnetic reference frames are unable to resolve absolute longitudinal motions and include true polar wander, which is unrelated to plate-mantle interactions^21^. Coastlines, boundaries between continental and ocean crust, plate polygons, and evolving boundaries (subduction, spreading, transform) were reconstructed at 10 Myr intervals.

### Craton boundaries and deposit data

Craton boundaries were mapped using an unsupervised machine learning approach applied to the REVEAL full-waveform seismic tomography model^13^, using horizontal shear wave velocity (V_sh_), vertical shear wave velocity (V_sv_), and isotropic P-wave velocity (V_p_) anomalies to delineate lithospheric roots. Age-coded mineral deposits were compiled from a recent study^5^, yielding a global inventory of 2166 deposits with classifications, ages, and contained metal resources.

Craton-related deposits from other thick lithosphere edge deposits were separated to focus specifically on craton-associated ore bodies. This distinction was made using a decision-tree approach, which allowed us to identify and isolate craton-related deposits, encompassing over 85% of the total metal content from the original database^5^. We chose this approach to better capture the relationship between craton edges and metallogenesis.

The boundaries of these cratons improved the previous work at detecting craton boundaries^5^, reducing the search space for craton-related deposits from 200 km to 90 km, resulting in more accurate craton edge identification^14^.

### Spatiotemporal analysis

For each 10 Myr time step, we reconstructed craton boundaries and deposit positions using pyGPlates/GPlately^36^—a Python library that builds on the plate reconstruction capabilities originally developed in GPlates^35^, and calculated distances from each deposit to (i) the nearest craton edge and (ii) the nearest subduction trench. To test whether deposits occupy a distinct tectonic setting, we generated 500 random points within a 185 km buffer around craton boundaries and propagated them through all 180 time steps, producing 90,000 random reference locations. Statistical comparisons used two-sample Kolmogorov–Smirnov tests^22^ and cumulative distribution functions to quantify differences between deposit and random-point distributions. We conduct spatio-temporal modelling by combining geological data with plate tectonic reconstructions.

### Numerical geodynamic model

We use a present-day plate velocity GPML file distributed with ASPECT. It is based on the EarthByte global rotation model for the present day only^37^. The rationale for using present-day GPlates-derived velocities is to ground the synthetic models in an internally consistent, observation-based plate kinematic framework rather than prescribing arbitrary convergence rates. While multiple plate boundaries exist in the annulus, our analysis focuses exclusively on the chosen subduction zone as the primary site of convergence relevant to this study. Other boundaries are present as part of the global kinematic system but are not the focus of interpretation.

We simulated mantle flow and lithospheric deformation using the community geodynamics code ASPECT^23^, which solves the conservation equations for mass, momentum, and energy using a finite element approach. The models were run in a 2D spherical annulus geometry with adaptive mesh refinement, using several hundred computational cores. The energy equation includes adiabatic, shear, and radiogenic heating terms. Boundary conditions consisted of a constant temperature of 2600 K at the core–mantle boundary, 273 K at the surface, and free-slip at the model base. Surface velocities were prescribed using present-day GPlates plate kinematic reconstructions to drive realistic subduction.

The convection equation set is:

$$\nabla \cdot {u}^{ \rightharpoonup }=-\left(\frac{1}{\rho }\frac{\partial \rho }{\partial p}\right)\rho g{u}^{ \rightharpoonup }$$

(Equation S1)

$$-\nabla \cdot \left[2\eta \left(\dot{{{\boldsymbol{\varepsilon }}}}\left({u}^{ \rightharpoonup }\right)-\frac{1}{3}\left(\nabla \cdot {u}^{ \rightharpoonup }\right){{\boldsymbol{I}}}\right)\right]+\nabla p=\rho {g}^{ \rightharpoonup }$$

(Equation S2)

$$\rho {C}_{p}\left(\frac{\partial T}{\partial t}+{u}^{ \rightharpoonup }\cdot \nabla T\right)-\nabla \cdot k\nabla T=\rho H+\alpha T\left({u}^{ \rightharpoonup }\cdot \nabla p\right)\, \\+2\eta \left(\dot{{{\boldsymbol{\varepsilon }}}}\left({u}^{ \rightharpoonup }\right)-\frac{1}{3}\left(\nabla \cdot {u}^{ \rightharpoonup }\right){{\boldsymbol{I}}}\right):\left(\dot{{{\boldsymbol{\varepsilon }}}}\left({u}^{ \rightharpoonup }\right)-\frac{1}{3}\left(\nabla \cdot {u}^{ \rightharpoonup }\right){{\boldsymbol{I}}}\right)\,$$

(Equation S3)

In this formulation ${u}^{ \rightharpoonup }$ is the velocity, $\rho$ density, p pressure, g gravitational acceleration, $\eta$ viscosity, $\dot{{{\boldsymbol{\varepsilon }}}}\left({{{\rm{u}}}}^{ \rightharpoonup }\right)=\frac{1}{2}\left(\nabla {u}^{ \rightharpoonup }+\nabla {{u}^{ \rightharpoonup }}^{T}\right)$ strain-rate tensor, Cp heat capacity, k thermal conductivity, H internal heating rate, and $\alpha$ thermal expansivity. The energy equation (Eq. S3) includes terms for shear heating, adiabatic heating, and radioactive heating.

We set an initial adiabatic temperature at atmospheric pressure to be 1600 K, with constant temperature boundary conditions at the core of 2600 K and at the surface of 273 K. The former has been set to suppress vigorous (and artificial) initial plume flow, which impacts the subduction-induced mantle velocities.

The initial surface velocities are imposed from the plate model by Zahirovic et al.^37^ from the GPML included in the ASPECT distribution. We utilise two points to define the plane through which the spherical annulus model passes. These are set to a colatitude of 1.5708 radians (for example, at the equator) for both points, with longitudes defined by 4.87 and 5.24 radians (279 and 300 degrees, these are arbitrary for an equatorial slice). Free slip velocities are imposed at the base.

We use a composite viscoplastic model comprising three deformation mechanisms: diffusion creep, dislocation creep, and yielding. This takes the form

$${{\rm{\eta }}}=1/\left(\frac{1}{{\eta }_{{diff}}}+\frac{1}{{\eta }_{{disl}}}+\frac{1}{{\eta }_{y}}\right)$$

(Equation 4)

Diffusion and dislocation creep viscosities are calculated using an Arrhenius form:

$${\eta }_{{diff}/{disl}}={A}^{-\frac{1}{n}}\exp \left(\frac{E+{pV}}{{nRT}}\right){{\dot{\varepsilon }}_{{II}}}^{\frac{1-n}{n}}$$

(Equation 5)

Here, the gas constant is R, A is a prefactor, n is the stress exponent, E is the activation energy, V is the activation volume, and ${\dot{\varepsilon }}_{{II}}$ is the second invariant of the strain rate tensor. These parameters change over the phase change, defining the upper to lower mantle transition. Yielding is simulated via an effective viscosity of the form^38^:

$${\eta }_{y}=\frac{{\tau }_{0}+{fp}}{2{\dot{\varepsilon }}_{{II}}}$$

(Equation 6)

Here, the surface yield strength is ${\tau }_{0}$, and the friction coefficient is f. The full list of parameters used in our models is shown in Table S1.

Table 1 summarises the physical and rheological parameters adopted in the numerical geodynamic simulations. The model represents the Earth with realistic dimensions and gravitational conditions, incorporating standard values for the core–mantle boundary temperature, thermal conductivity, expansivity, and heat capacity. Distinct rheological properties are prescribed for the upper mantle, transition zone, lower mantle, and continental lithosphere, with different prefactors and activation parameters for both diffusion and dislocation creep mechanisms to capture depth-dependent variations in viscosity. Diffusion creep is characterised by a linear stress–strain relationship, while dislocation creep exhibits a non-linear response reflecting strain-rate sensitivity. The model also incorporates a brittle yield criterion, defined by surface yield strength and frictional parameters, enabling the simulation of lithospheric failure. Phase transitions associated with mantle mineral transformations are implemented at depths corresponding to major seismic discontinuities, each represented by a finite width to ensure smooth transitions. Layer-dependent densities are specified to account for compositional and thermal variations within the mantle and lithosphere.

**Table 1:** Numerical geodynamic simulation parameters

Earth radius	R (km)	6371
Core radius	Rc (km)	3481
Gravity acceleration	G (m/s2)	9.81
Initial CMB temperature	TCMB (K)	2600
Thermal conductivity	K (W/K)	4.7
Thermal expansivity	a (W/m.K)	2e-5
Heat capacity (all)	C (J/K)	1250
Pre-factor, diffusion creep (upper mantle |transition zone | lower mantle | continent)	A (Pa-ns-1)	6e-17 | 9e-17 | 1e-18 | 6e-20
Pre-factor, dislocation creep (upper mantle |transition zone | lower mantle | continent)	A (Pa-ns-1)	6.51e-16 | 8.51e-16 | 6.51e-16 | 6.51e-28
Activation energy, diffusion creep (upper mantle |transition zone | lower mantle | continent)	E (kJ/mol)	150e3 | 155e3 | 150e3, 166e3
Activation energy, dislocation creep (upper mantle |transition zone | lower mantle | continent)	E (kJ/mol)	500e3 | 500e3 | 530e3, continent: 540e3
Activation volume, diffusion creep (upper mantle |transition zone | lower mantle | continent)	V (cm3/mol)	6.34e-7 | 14.34e-7 | 12.34e-7 | 6.34e-7
Activation volume, dislocation creep (upper mantle |transition zone | lower mantle | continent)	V (cm3/mol)	1.3e-5 | 1.3e-5 | 1.3e-5 | 18e-6
Stress exponent, diffusion creep (upper mantle |transition zone | lower mantle | continent)	n	1 | 1 | 1 | 1
Stress exponent, dislocation creep (upper mantle |transition zone | lower mantle | continent)	n	3.0 | 3.0 | 1.0 | 1.0
Surface yield strength	τ0 (MPa)	20
Friction coefficient***	f	0.2
Phase transition depths	d (km)	410, 600
Phase transition widths	w (km)	50, 50
Densities (upper mantle |transition zone | lower mantle | continent)	r (kg/m3)	3300 | 3500 | 3800 | 2900

The lithosphere–asthenosphere system was modelled as a composite viscoplastic material, with effective viscosity determined by the combined contributions of diffusion creep, dislocation creep, and yielding. Viscosity variations follow an Arrhenius law parameterised by activation energy, activation volume, and stress exponent, with distinct values for the upper mantle, transition zone, lower mantle, and continental material. Yielding was implemented using a Drucker–Prager criterion with a surface yield strength of 20 MPa and a friction coefficient of 0.2.

Initial conditions consisted of three continental blocks and two cratonic roots, imposed as chemically distinct regions with a lithospheric thickness of up to 150 km for continents and 300 km for cratons. The angular distance between the craton edges and the subduction zone was systematically varied (5°, 10°, 15°, 20°, and 30°) to represent different geodynamic configurations, corresponding to trench–craton distances between ~700 km and ~2800 km. Each model was evolved for 27 Myr to allow the system to reach quasi-steady-state flow. Model outputs include temperature, strain rate, and stress fields, with strain and stress maxima extracted along craton edges to quantify the effect of subduction-driven mantle flow on lithospheric weakening. Subcontinental strain-rate and the second deviatoric stress tensor were integrated between depths of 100–300 km to give horizontal profiles (Fig. 4), and peak estimates from these profiles were compiled in Fig. 5.

Our geodynamic simulations are designed as simplified physical experiments that capture the first-order interaction between subduction-driven return flow and a strong cratonic lithosphere; they are not intended to reproduce detailed paleo-geometries, time-specific lithospheric thicknesses, or the evolving configuration of individual craton edges. This approach is appropriate because cratonic interiors and many craton edges have remained relatively stable and long-lived through time—as evidenced by the preservation of major sediment-hosted deposits in these settings—despite local modification or reworking in some regions.

The detailed numerical implementation, including mantle layering, thermal structure, and the exact angular positions of continental blocks and cratonic roots, is defined using a World-Builder configuration file and is provided in the Supplementary Material for full reproducibility.

### Workflow

**Fig. 6:**
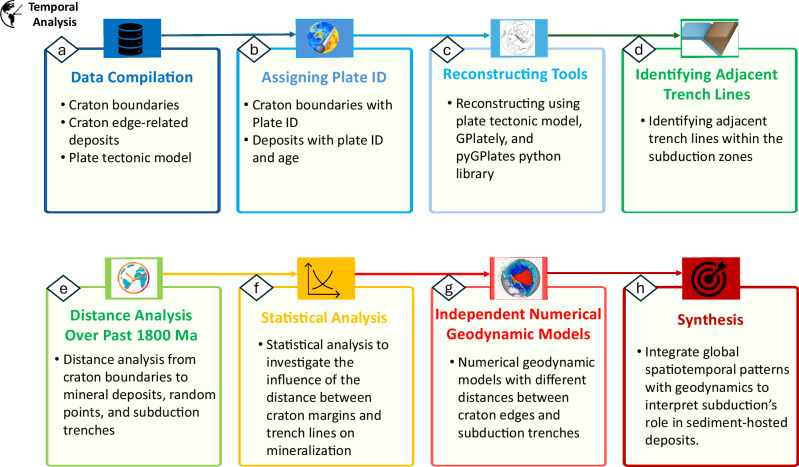
Workflow of linking subduction zones to craton-edge sediment-hosted deposits. **a** Data compilation of craton boundaries, craton edge-related deposits, and the plate tectonic model. **b** Assigning plate IDs to craton boundaries and deposits (with age). **c** Reconstructing data using a plate tectonic model, GPlately, and the pyGPlates Python library. **d** Identifying adjacent trench lines within the subduction zones. **e** Distance analysis over the past 1800 Ma from craton boundaries to mineral deposits, random points, and subduction trenches. **f** Statistical analysis investigating the influence of the distance between craton margins and trench lines on mineralization. **g** Independent numerical geodynamic models with different distances between craton edges and subduction trenches. **h** Synthesis integrating global spatiotemporal patterns with geodynamics to interpret subduction's role in sediment-hosted deposits.

The workflow begins with data synthesis (Step a in Fig. 6), in which craton boundaries, near-craton mineral deposits, and a global plate tectonic model are compiled. Plate IDs are then assigned to craton boundaries and deposits according to their ages (Step b). Using GPlately and pyGPlates³⁸, craton boundaries and deposits are reconstructed through time to ensure accurate temporal positioning (Step c), followed by identification of subduction trench lines adjacent to cratons at each time step (Step d). Distances from each deposit and a set of randomly generated points to the nearest craton edge and subduction trench are then calculated based on the plate configuration at the time of formation (Step e). Statistical analyses compare the distance distributions of deposits and random points to assess whether mineralised craton edges are preferentially associated with proximity to subduction zones (Step f). Numerical geodynamic models with varying craton–trench configurations are used to test the influence of craton–trench distance (Step g). Finally, global spatiotemporal patterns are integrated with geodynamic modelling results to evaluate how subduction influences the formation of sediment-hosted mineral deposits (Step h). This streamlined workflow isolates and quantifies the effect of craton–trench distance on mineralisation, providing a robust framework for evaluating the geodynamic setting of mineral deposit formation.

**Fig. 7:**
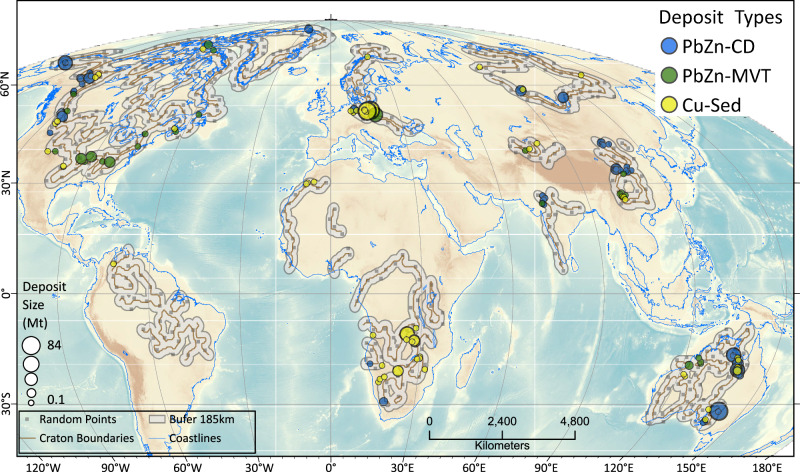
Location of 500 randomly generated points across 180 time steps (1 to 1800 Ma at 10 Ma Intervals). A total of 90,000 random points were generated (500 points × 180 time steps), spanning from 1 Ma to 1800 Ma in 10-million-year intervals. Given that over 90% of all craton-hosted deposits are located within a 185 km buffer of craton boundaries^14^. All random points were generated within this buffer zone.

The spatial distribution of 500 randomly generated points at the present day is illustrated in Fig. 7. These random points were also generated across 180 time steps, spanning from 1 to 1800 Ma in 10-million-year intervals, resulting in a total of 90,000 points (500 points × 180 steps). To ensure geological relevance and to explore why mineral deposits tend to concentrate in certain parts of craton edges over time, all points were generated within a 185 km buffer of craton boundaries. This constraint is based on the observation that over 90% of known craton-hosted deposits fall within this distance^14^. By focusing on this zone, the approach aims to investigate spatiotemporal patterns of potential mineralisation through deep time.

## Supplementary information

Supplementary Information

Description of Additional Supplementary Files

Movie_S1

Movie_S2

Transparent Peer Review file

## Data Availability

The Craton boundaries, mineral systems data used in this study, have been deposited in the link below. https://github.com/EarthByte/Craton-deposits_Tectonic-evolution.

## Code availability

Python scripts, the optimised mantle reference frame plate tectonic model, and ASPECT codes are available in the following archive: 10.5281/zenodo.15015718.
