# Supplementary material for: How subduction evolution drives sediment-hosted mineralisation along craton edges
Source: Nat Commun. 2026 Jun 10;17:7367. doi: 10.1038/s41467-026-74134-5 (PMC13402583; doi:10.1038/s41467-026-74134-5)
Supplement: Supplementary file 1 — Supplementary Information [file 41467_2026_74134_MOESM1_ESM.pdf]

Supplementary information:

Auxiliary Geodynamic Results

Model examples after 27 Ma of subduction, for different initial distances between the subduction zone and the craton. Colours represent temperature (blue–red divergent colours) and strain rate (variable transparency inferno colour scale – purple–yellow). Arrows represent (non-scaled) velocity directions. The green line is a contour of the continental/cratonic material.

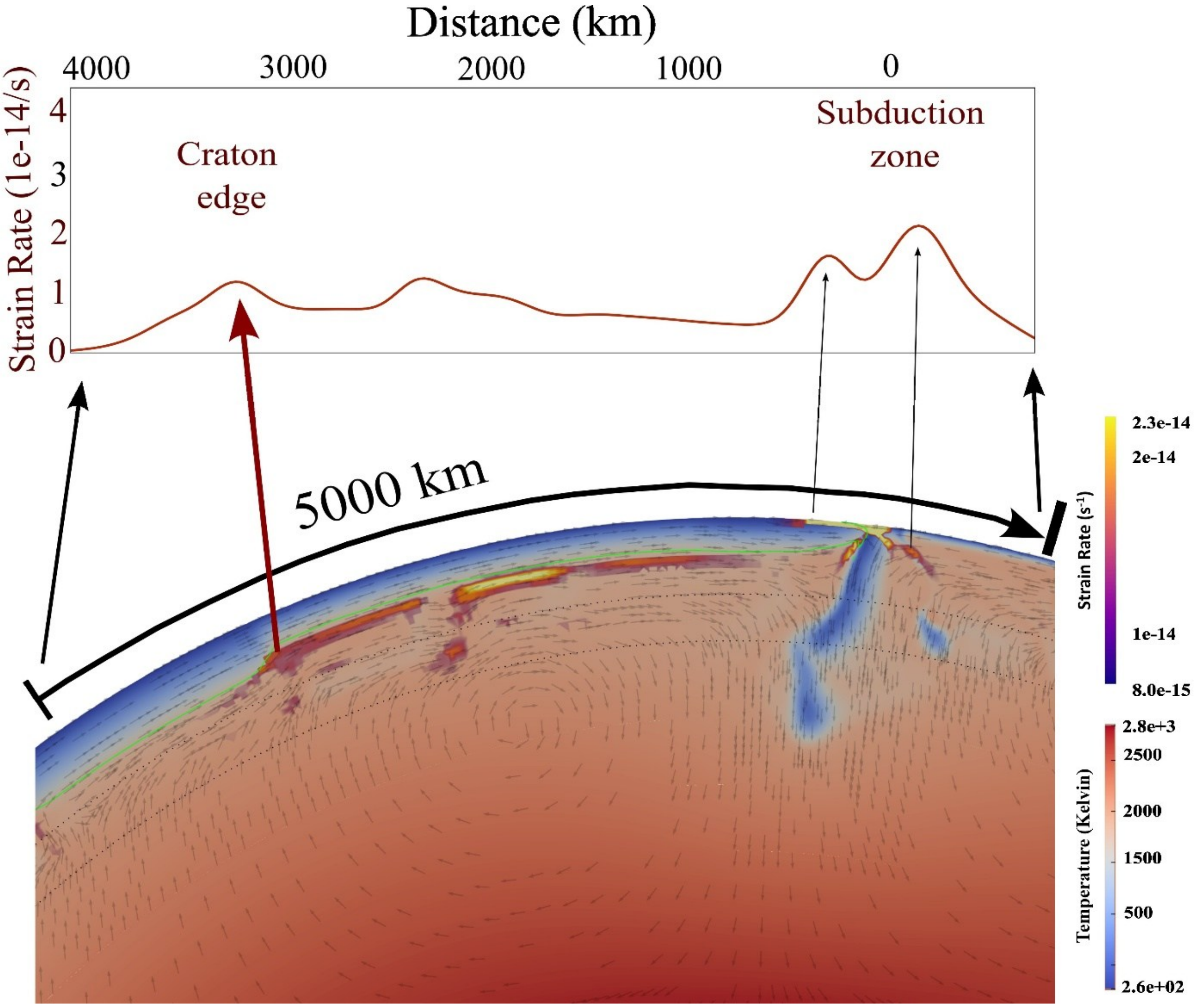

Fig. S1. Numerical model of subduction-driven mantle return flow with a high peak at trench–craton distance of ~3,400 km. Equi-length flow arrows show a broad subduction-related flow cell extending >5,000 km from the trench, with vertical flow velocities focused near the craton edge. Peak subcontinental strain rates occur at the craton edge, illustrating how subduction localises deformation far into the overriding plate.

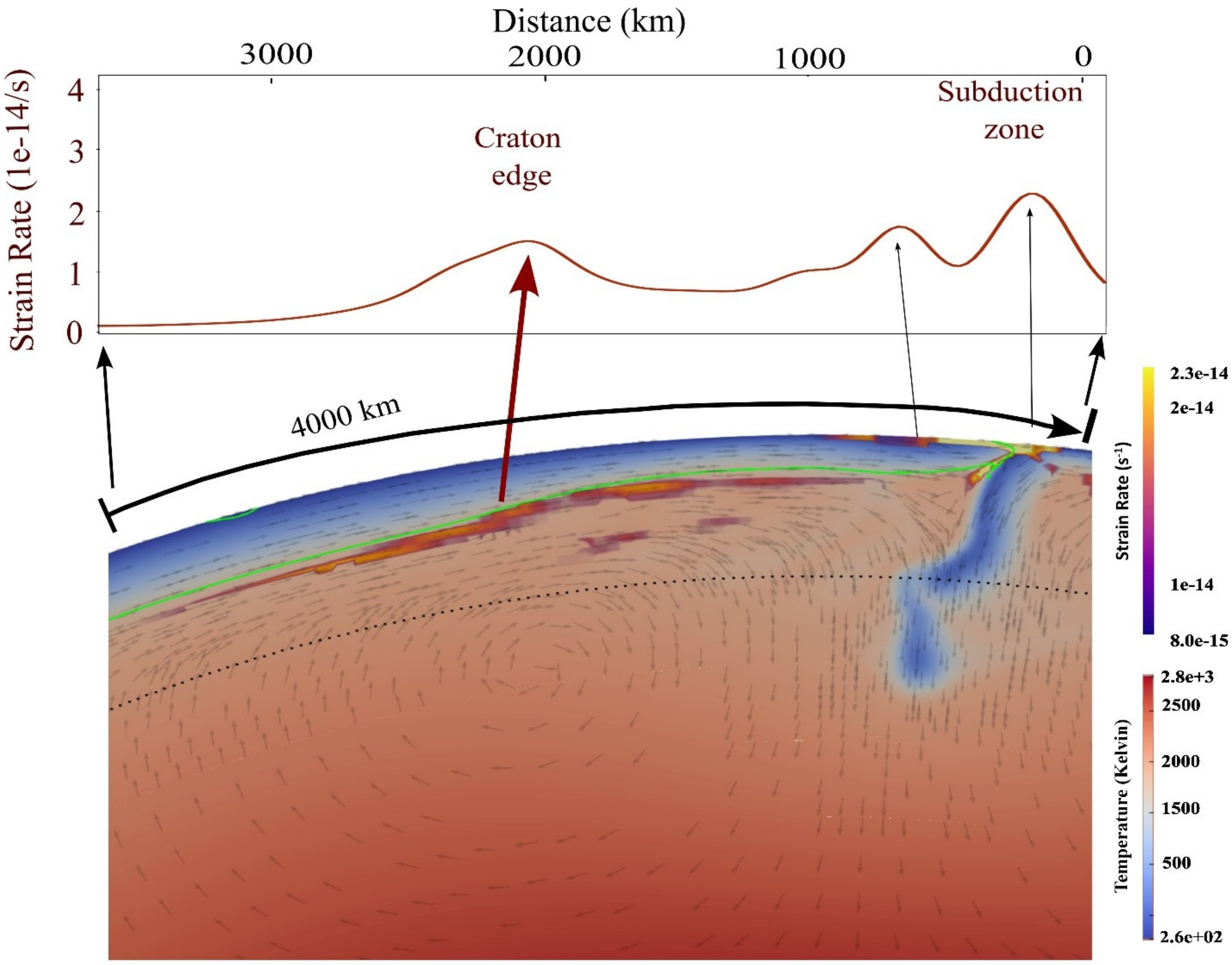

Fig. S2. Numerical model of subduction-driven mantle return flow with a high peak at trench–craton distance of ~2,300 km.. Equi-length flow arrows show a broad subduction-related flow cell extending >4,000 km from the trench, with vertical flow velocities focused near the craton edge. Peak subcontinental strain rates occur at the craton edge, illustrating how subduction localises deformation far into the overriding plate.

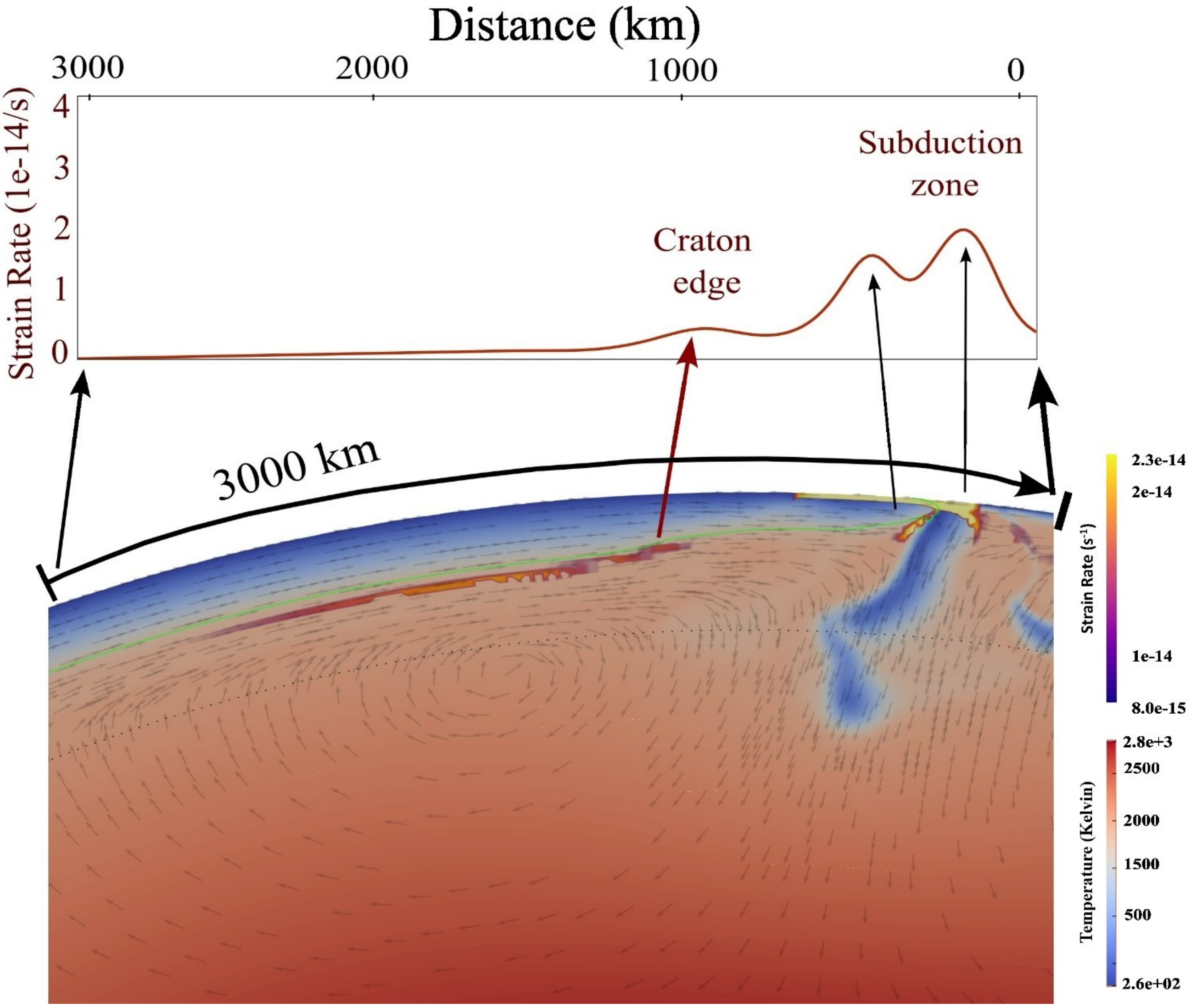

Fig. S3. Numerical model of subduction-driven mantle return flow with a high peak at trench–craton distance of ~900 km. Equi-length flow arrows show a broad subduction-related flow cell extending >3,000 km from the trench, with vertical flow velocities focused near the craton edge. Peak subcontinental strain rates occur at the craton edge, illustrating how subduction localises deformation far into the overriding plate.

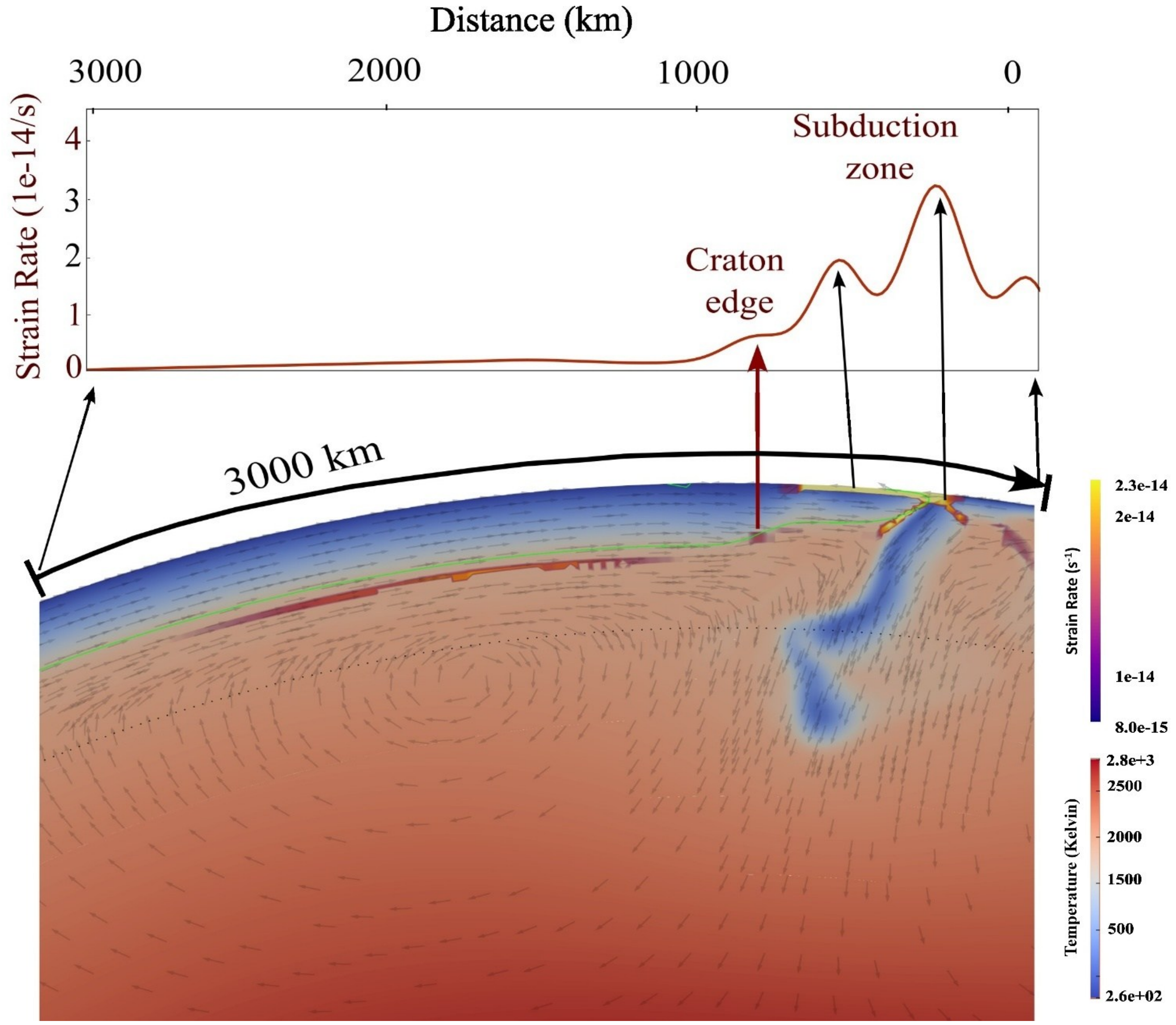

Fig. S4. Numerical model of subduction-driven mantle return flow with a high peak at trench–craton distance of ~600 km. Equi-length flow arrows show a broad subduction-related flow cell extending >4,000 km from the trench, with vertical flow velocities focused near the craton edge. Peak subcontinental strain rates occur at the craton edge, illustrating how subduction localises deformation far into the overriding plate.

Table S1: Summary of results from simulations I-V above. Results calculated for 27 Myr (timeslot shown). Angular distance refers to the hemispheric angular distance between the subduction zone and the cratonic margin; this is also given in a great arc distance. Craton edge strain and stress represent the maximum of these values at the cratonic gradient, as shown in the graphs in Figs. S1- to S4 and Fig 5 in the paper.

Simulation	Angular distance	Distance (km)	Craton edge strain (1e-14/s)	Stress (1e9)
30	25.28	3400	1.3	0.8
20	21.1413	2300	1.5	0.8
15	17.65	1300	2.1	1.3
10	12.238	900	0.6	0.9
5	6.3756	600	0.7	1

Model Description

We use the community geodynamics code *Aspect*[1]. The code solves the basic conservation equations for mantle convection using a finite element approach. We utilise adaptive grid refinement in a 2D spherical annulus, utilizing several hundred cores. The convection equation set is:

$$\nabla \cdot \vec{u} = -(\frac{1}{\rho}\frac{\partial \rho}{\partial p})\rho g \vec{u}$$

(Equation S1)

$$-\nabla \cdot \left[2\eta\left(\dot{\boldsymbol{\varepsilon}}(\vec{u}) - \frac{1}{3}(\nabla \cdot \vec{u})\boldsymbol{I}\right)\right] + \nabla p = \rho \vec{g}$$

(Equation S2)

$$\rho C_p\left(\frac{\partial T}{\partial t} + \vec{u} \cdot \nabla T\right) - \nabla \cdot k \nabla T = \rho H + \alpha T(\vec{u} \cdot \nabla p) \; + 2\eta\left(\dot{\boldsymbol{\varepsilon}}(\vec{u}) - \frac{1}{3}(\nabla \cdot \vec{u})\boldsymbol{I}\right) : \left(\dot{\boldsymbol{\varepsilon}}(\vec{u}) - \frac{1}{3}(\nabla \cdot \vec{u})\boldsymbol{I}\right)$$

(Equation S3)

In this formulation $\vec{u}$ is the velocity, ρ density, p pressure, g gravitational acceleration, η viscosity, $\dot{\boldsymbol{\varepsilon}}(\vec{u}) = \frac{1}{2}(\nabla \vec{u} + \nabla \vec{u}^T)$ strain-rate tensor, *Cp* heat capacity, *k* thermal conductivity, *H* internal

heating rate, and α thermal expansivity. The energy equation (Eq S3) includes terms for shear heating and adiabatic heating, and radioactive heating.

We set an initial adiabatic temperature at atmospheric pressure to be 1600K, with constant temperature boundary conditions at the core of 2600 K and at the surface of 273K. The former has been set to suppress vigorous (and artificial) initial plume flow, which impact the subduction-induced mantle velocities.

The surface velocities are set by present-day gplates velocities, from the gpml included in the Aspect distribution. It is based on the Earthbyte global rotation model, for the present day only. The relevant reference is : *Zahirovic, S., Eleish, A., Doss, S., Pall, J., Cannon, J., Pistone, M., and Fox, P., 2022, Subduction kinematics and carbonate platform interactions: Geoscience Data Journal, doi: 10.1002/gdj3.146.*

We utilize two points to define the plane through which the spherical annulus model passes. These are set to a colatitude of 1.5708 radians (ie. at the equator) for both points, with longitudes defined by 4.87 and 5.24 radians (279 and 300 degrees, these are arbitrary for an equatorial slice). Free slip velocities are imposed at the base.

We use a composite visco-plastic comprised of three deformation mechanisms: diffusion creep, dislocation creep, and yielding. This takes the form

$$\eta = 1/(\frac{1}{\eta_{diff}} + \frac{1}{\eta_{disl}} + \frac{1}{\eta_y})$$

(Equation 4)

Diffusion and dislocation creep viscosities are calculated using an Arrhenius form:

$$\eta_{diff/disl} = A^{-\frac{1}{n}} \exp\left(\frac{E + pV}{nRT}\right) \dot{\varepsilon}_{II}^{\frac{1-n}{n}}$$

(Equation 5)

Here, the gas constant is R, A is a prefactor, n the stress exponent, E is the activation energy, V the activation volume, and $\dot{\varepsilon}_{II}$ the second invariant of the strain rate tensor. These parameters change over the phase change, defining the upper to lower mantle transition. Yielding is simulated via an effective viscosity of the form[2]:

$$\eta_y = \frac{\tau_0 + fp}{2\dot{\varepsilon}_{II}}$$

(Equation 6)

Here the surface yield strength is τ_0, and the friction coefficient is *f*. The full list of the parameters used in our models is shown in Table S1 below.

Table S2. Simulation parameters

Earth radius	R (km)	6371
Core radius	R_c (km)	3481
Gravity acceleration	g (m/s^2)	9.81
Initial CMB temperature	T_{CMB} (K)	2600
Thermal conductivity	K (W/K)	4.7
Thermal expansivity	α (W/m.K)	2e-5
Heat capacity (all)	C (J/K)	1250
Pre-factor, diffusion creep (upper mantle |transition zone | lower mantle | continent)	A (Pa^{-n}s^{-1})	6e-17 | 9e-17 | 1e-18 | 6e-20
Pre-factor, dislocation creep (upper mantle |transition zone | lower mantle | continent)	A (Pa^{-n}s^{-1})	6.51e-16 | 8.51e-16 | 6.51e-16 | 6.51e-28
Activation energy, diffusion creep (upper mantle |transition zone | lower mantle | continent)	E (kJ/mol)	150e3 | 155e3 | 150e3 , 166e3
Activation energy, dislocation creep (upper mantle |transition zone | lower mantle | continent)	E (kJ/mol)	500e3 | 500e3 | 530e3 , continent: 540e3
Activation volume, diffusion creep (upper mantle |transition zone | lower mantle | continent)	V (cm^3/mol)	6.34e-7 | 14.34e-7 | 12.34e-7 | 6.34e-7
Activation volume, dislocation creep (upper mantle |transition zone | lower mantle | continent)	V (cm^3/mol)	1.3e-5 | 1.3e-5 | 1.3e-5 | 18e-6
Stress exponent, diffusion creep (upper mantle |transition zone | lower mantle | continent)	n	1 | 1 | 1 | 1
Stress exponent, dislocation creep (upper mantle |transition zone | lower mantle | continent)	n	3.0 | 3.0 | 1.0 | 1.0
Surface yield strength	τ_0 (MPa)	20
Friction coefficient***	f	0.2
Phase transition depths	d (km)	410, 600
Phase transition widths	w (km)	50, 50
Densities (upper mantle |transition zone | lower mantle | continent)	ρ (kg/m^3)	3300 | 3500 | 3800 | 2900

The parameters were adapted from O'Neill (2020)[3] and O'Neill et al. (2018)[4], and adjusted to give reasonable slab viscosities, slab velocities, and upper mantle viscosities for the model configuration. We note that the timing of subduction return flow, and the length scales associated with it, are associated with these parameters, and we have aimed to construct an upper mantle with a comparable rheology to Earth. The (undeformed, non-plastic) slab viscosities in our models vary from 1e24-6e24 Pa.s^{-1}, the cratonic viscosity varies from 2.6e21 – 3.4e24 Pa.s^{-1}, and the upper mantle viscosities vary from 1.7 e21 Pa.s^{-1} beneath the craton, to 3.7e21 Pa.s^{-1} in the transition zone. These values are in line with geoid and postglacial rebound constraints[5].

The initial model configuration is described in a World-Builder file, and includes mantle material to all depths, divided into the linear layers (0 – 10 km, 300 → 300K; 10 – 120 km, 300 →1700 K; 120 – 2700 km, 1700 – 2700 K; 2700 – 2895 km, 2700 – 2700 K). Three continental rafts and two cratonic roots are then imposed. The continents are imposed at 0 – 27 degree, 85 – 155 degrees, and 260 – 307 degrees, to a depth (ramped) of 150 km. The cratonic root extents are varied over each run, but (for example) run from 117 – 135 degrees (the 117 longitude is varied in the control experiments) and 267 – 287 degrees, to 300 km depth. The cratons have identical properties to the continents in these simulations.

Time Series

The following plots show a time series of one simulation, with a craton at approximately 20° from the subduction zone initially.

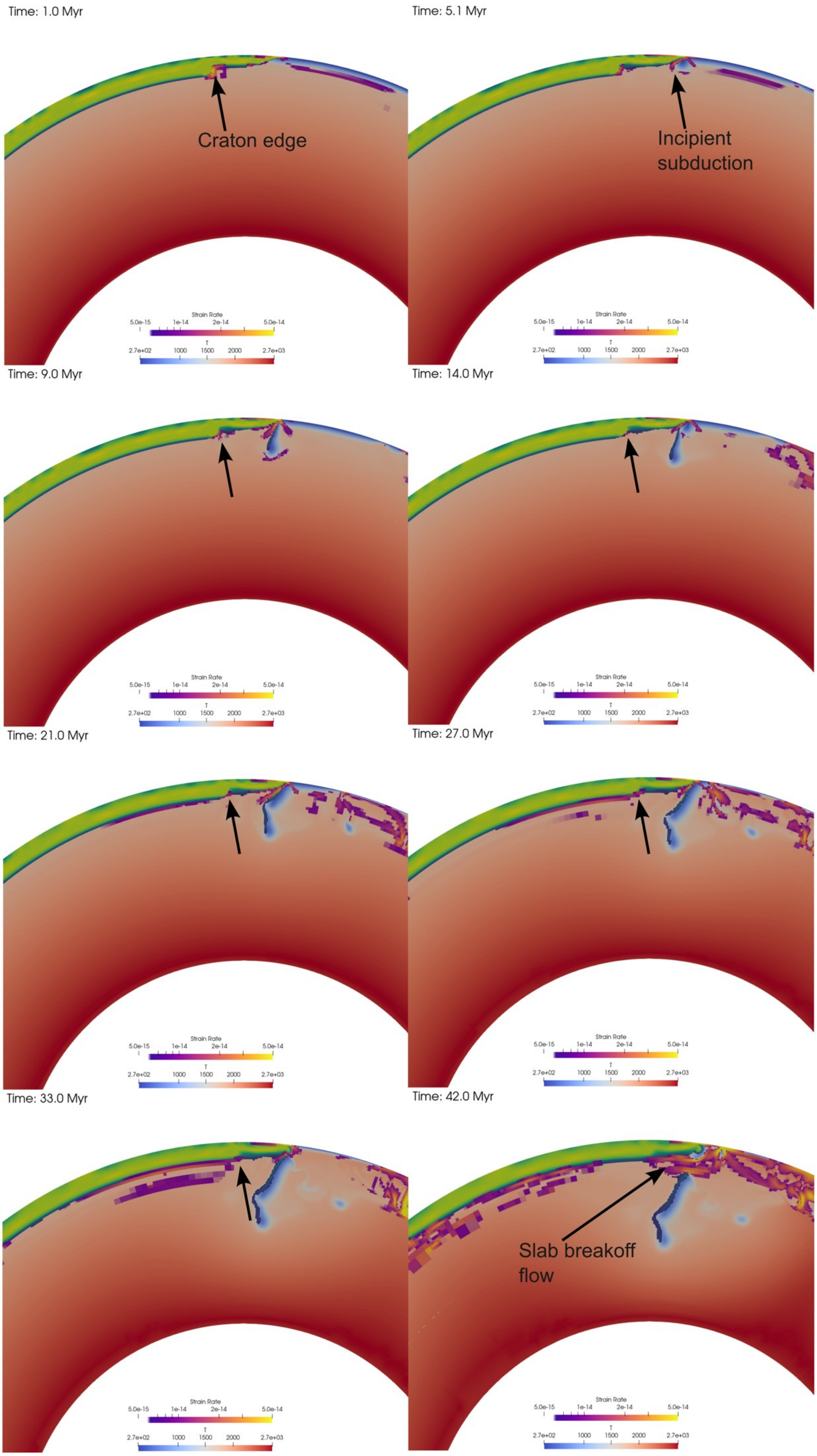
Time: 1.0 Myr
Craton edge
Time: 5.1 Myr
Incipient
subduction
Time: 9.0 Myr
Time: 14.0 Myr
Time: 21.0 Myr
Time: 27.0 Myr
Time: 33.0 Myr
Time: 42.0 Myr
Slab breakoff
flow
Strain Rate
5.0e-15 1e-14 2e-14 5.0e-14
T
2.7e+02 1000 1500 2000 2.7e+03

Fig. S5.: Time series of the evolution of a subduction zone, initially at a distance of 20 degrees from a cratonic boundary. The subduction zone evolves from incipient subduction to a mature subducting slab through to slab breakoff over 42 Myr. Over this period, the cratonic margin illustrates consistently high localized strain until the slab breakoff event.

Auxiliary Citations

Kronbichler, M., Heister, T. and Bangerth, W., 2012. High accuracy mantle convection simulation through modern numerical methods. *Geophysical Journal International*, *191*(1), pp.12-29.

Moresi, L. and Solomatov, V., 1998. Mantle convection with a brittle lithosphere: thoughts on the global tectonic styles of the Earth and Venus. *Geophysical Journal International*, *133*(3), pp.669-682.

O'Neill, C., 2020. Planetary thermal evolution models with tectonic transitions. *Planetary and Space Science*, *192*, p.105059.

O'Neill, C.J. and Zhang, S., 2018. Lateral mixing processes in the Hadean. *Journal of Geophysical Research: Solid Earth*, *123*(8), pp.7074-7089.

O'Neill, C. and Aulbach, S., 2022. Destabilization of deep oxidized mantle drove the Great Oxidation Event. *Science Advances*, *8*(7), p.eabg1626.
