# Supplementary material for: How subduction evolution drives sediment-hosted mineralisation along craton edges
Source: Nat Commun. 2026 Jun 10;17:7367. doi: 10.1038/s41467-026-74134-5 (PMC13402583; doi:10.1038/s41467-026-74134-5)
Supplement: Supplementary file 2 — Description of Additional Supplementary Files [file 41467_2026_74134_MOESM2_ESM.pdf]

Description of Additional Supplementary Files

Supplementary Movie S1- This video shows the reconstructed positions of craton-edge mineral deposits through geological time, together with craton edges, continents, and trench lines. Deposit symbol size is proportional to metal endowment, ranging from 0.1 to 84 Mt. The animation highlights the evolving relationship between mineralisation and subduction through time.

Supplementary Movie S2- This video shows a numerical geodynamic simulation of subduction-driven mantle flow beneath a craton. Mantle return flow generates focused vertical velocities and elevated strain and stress rates near the craton edge, demonstrating how subduction can localise deformation far into the overriding plate. The lithosphere–asthenosphere boundary (LAB), marked by the green line.
