# Supplementary material for: How subduction evolution drives sediment-hosted mineralisation along craton edges
Source: Nat Commun. 2026 Jun 10;17:7367. doi: 10.1038/s41467-026-74134-5 (PMC13402583; doi:10.1038/s41467-026-74134-5)
Supplement: Supplementary file 5 — Transparent Peer Review file [file 41467_2026_74134_MOESM5_ESM.pdf]

nature portfolio

Peer Review File

How Subduction Evolution Drives Sediment-Hosted Mineralisation Along Craton Edges

Corresponding Author: Mr Hojat Shirmard

This file contains all reviewer reports in order by version, followed by all author rebuttals in order by version.

Version 0:

Reviewer comments:

Reviewer #1

(Remarks to the Author)
Review of the manuscript "How Subduction Evolution Drives Sediment-Hosted Mineralisation Along Craton Edges" by Shirmard et al. submitted to nature communications.
This study comprises a two-step investigation. Firstly, the authors find that the metallogenesis clusters at the craton margins that are 800–1,800 km from subduction zones, based on the global plate motion model and a global mineral deposit database. Then, several 2D geodynamic models have been conducted with ASPECT software, which demonstrate that locations of elevated stress and strain along craton edges coincide with subduction-driven mantle return flow at similar distances from subduction zones. Finally, they propose that subduction may play a first-order control on craton-margin mineralisation, based on the inference that "mantle return flow weakens craton margins, promotes rifting, channels fluids into the crust, enriching the lithosphere with volatiles, and fostering metallogenesis".
The paper is well-targeted and clearly illustrated. My main concern lies in the (weak) connection between simple numerical models and the complex process of metallogenesis.
(1) I am not very convinced by the relationship between the modelled relatively high strain rate at the bottom of craton edge and the metallogenesis in the shallow crust.
Firstly, the high strain rate is widely distributed beneath the continental plate (e.g. in Figure 5), although a peak is predicted at the craton edge by integrating vertically between 100–300 km. I am curious about the vertical integration; why is 100–300 km used. The lithosphere thickness of craton is 300 km, but that of thin continental plate is up to 150 km. Thus, if you integrate between 100–300 km, the high strain rate region beneath craton will not be included.
Secondly, I am wondering how to connect the relatively high strain rate beneath the continental lithosphere (induced by mantle flow shearing) to the shallow crustal deformation and metallogenesis. It is inferred that "mantle return flow weakens craton margins, promotes rifting, channels fluids into the crust, enriching the lithosphere with volatiles, and fostering metallogenesis". However, all these processes are not predicted in the present numerical models; the continental lithosphere in these models is kind of rigid without any strain localization (Figures 5, and S1–S4).
(2) The observed deposit distance from subduction trench is 800–1800 km, whereas the numerically modelled strain maxima beneath the continent is 1500–2500 km. The authors argue that they are slight offset and attribute this discrepancy to the possible inherited lithospheric heterogeneities and 3D mantle flow patterns that are not considered in the present model. In my opinion, this offset is not small enough to be neglected. I would suggest improving the model, e.g. including the factors as proposed, to find the controlling factor for such a trend of metallogenesis.
(3) By the way, the subducting slab-induced mantle flow pattern is strongly controlled by the length and angle of sinking slab. In this sense, it is time-dependent. In this study, a constant model time of 27 Ma is used. Although it is argued that it reaches the steady-state, I am kind of curious about that, since the slab continues sinking and deforming. I would suggest plotting some time-dependent snapshots to check whether the high strain rate always localizes beneath the craton edge.
As a summary, I agree that the story is big and attractive; however, it requires many improvements to build the 'solid' connection between subduction-induced mantle flow and the crustal-scale metallogenesis, rather than a bunch of inferences.

Some minor points:
---The "MATERIALS AND METHODS" is organized rather loosely, for example, with many short sections and repeated contents.
---Lines 317-318, I do not think Fig. 3 and Fig. 4 are correctly cited.
---Lines 401-408, I do not know why the initial model configuration appears here, instead of in the section of "Numerical

geodynamic model".

(Remarks on code availability)

Reviewer #2

(Remarks to the Author)
In this study, the authors explore the potential geodynamic reasons behind the observation that major sediment-hosted base metal deposits are found on some (but not all) craton margins. They build their own database of craton margins and use a plate reconstruction to demonstrate that mineralised margins are likely to occur when there is a distance of 800-1800 km between that margin and a subduction zone. They then use 2-D geodynamic models of subduction zones to demonstrate that craton margins become sites of strain localisation at roughly this distance from trenches. They use these combined pieces of information to suggest that localised weakening of the sub-continental lithosphere at these locations provides optimal conditions for metasomatism, providing a means of boosting their fertility and preconditioning the location for mineral deposit formation.

I enjoyed reading your study and think it adds some interesting additional insights into this widely known observation. In my opinion, this material is worthy of publication in a journal with the stature of Nature Communications. Nevertheless, I have a number of points that need to be addressed before I would approve it for publication.

General points:

Enhanced predictability: The premise of this study is to improve our ability to predict deposit locations and this point is central to your conclusions. But where is the predictability? There doesn't seem to be a map of which of the present-day boundaries should be more or less prospective based on your results. But this aspect is likely to be the study's most useful output if the knowledge is going to be translated into exploration workflows.

Craton margins: You have used an AI-based approach to map the craton boundaries that are essential to your study, but have provided very scant details on the procedure and how well the model has performed. For starters, I do not think that I could remotely repeat this process. How does it work? What challenges did you need to think about to make it work? Is it possible that these are the same ones from your Economic Geology paper? If so you should certainly cite it! Secondly, how well has the algorithm done? There's no real comparison with other models and you should at least repeat the CDF of deposits versus random locations in the continents for this present-day setup to demonstrate that these boundaries are as good as (or better) than previous models. There is one brief mention in the Figure 8 caption of an SGTSG abstract, but that is insufficient evidence for a reader to pass judgement on these boundaries.

Deposits: I'm a bit concerned about your deposit databases. The ones you show in Figure 1 (top-left panel) and Figure 8 are not consistent with one another. And both are missing some major deposits that are in the databases of Hoggard et al., which is stated as where this database is taken from. For example, none of the major deposits in the Middle East show up. It is important that you explain these differences to avoid being accused of cherry-picking data. I personally don't like any removal of deposits from the original database if you are then generating CDFs and making statements about doing better than the original Hoggard et al. (2020) study. That would not be a fair comparison and you would need to perform the same analysis on the Hoggard et al. LAB maps.

Language: The manuscript can sometimes be quite repetitive and jumps back and forth. For example, you start the Geodynamic Model Results section with a sentence stating the results (lines 164-165), but this is then repeated on lines 189-191. You also have a statement in lines 169-170 about reasons for differences between your models and observations, but then different reasons are given in lines 220-223. Lines 229-235 seem to potentially say the same thing twice. The key ligands listed are different in Lines 218 and 241. The Materials and Methods section covers the craton boundaries and deposit databases in two places. The numerical modelling also seems to be split into two sections in different places. Apologies if I'm missing something, but this section needs to be appropriately streamlined and the rest of the text tidied up.

Specific points:

Introduction: Your study's motivation and methods seem to be heavily inspired by the study of Hoggard et al., (2020). It might be good to actually cite it somewhere in the main text? Perhaps in the introduction?

Figure 1: If your causal mechanism is about slabs fertilising the lithosphere prior to deposit genesis, why not show the deposit locations at periods prior to their formation time? These panels are also very small! And the time labels are not clearly associated with their respective panels (e.g. they are closer to the panel above).

Figure 3: This figure is interesting! But the caption is very brief and uninformative. Also what are the units of the density contours? I'm also not sure about your conclusion that deposits are more likely to be on cratons undergoing trench advance compared to other locations – whilst they do have negative trench-normal motion, it is less extreme than for the random locations (based on the density contours). Some might therefore argue that you want a less strong trench advance?

Figure 4: Sorry, but this figure is not so good. It is hard to interpret the information, there is no key for the colours, and much

of the information has already been shown in Figure 3. Can you perhaps make the three deposit classes into three different shaped symbols in Figure 3 and then colour them by age? Then you could drop this Figure 4.

Lines 168-170. None of these reasons seem to explain a systematic under-prediction of the distance?

Figure 5: This figure is important. Can you make the flow arrows more visible (they are too faint). Also don't put the two scale bars on top of the domain. The y-axis labels on the upper panel need appropriate formatting.

Figure 6: Why not plot this result for all five of the individual models? There are also typos in the axis labels and the units need formatting.

Line 316: Referencing error.

Line 329: Here you refer to IOCG deposits, but I don't see them elsewhere in the study?

Line 361: What is the rationale for driving these 2-D synthetic subduction models with real present-day plate velocities from GPlates? Where are they derived from? Does that mean the trench is not the only site of convergence/divergence?

Table 1: Where have these numbers come from or what is the justification for choosing them?

Figure 7: Thank you – this is a helpful figure for summarising your workflow.

Figure 8: Given these craton boundaries are a central component of your study, you should justify why you have made them as opposed to using previously published ones. This links back to my previous comment about explaining where these new ones come from. You need to demonstrate that your workflow is believable and does provide a good present-day match to the deposits (in my opinion, including those currently missing from this figure!). I think that this figure should also be in the main text (probably as Figure 1?).

(Remarks on code availability)

Reviewer #3

(Remarks to the Author)
This manuscript addresses an important and actively debated question in economic geology: why sediment-hosted metal deposits preferentially develop along craton margins. The integration of global plate reconstructions, seismic-defined craton boundaries, metal deposit databases, statistical analyses and numerical geodynamic simulations is ambitious and has the potential to generate meaningful insights. Shirmard et al. (2025) proposes a unifying geodynamic framework in which subduction-driven mantle return flow promotes lithospheric weakening and enhances metallogenic fertility along craton edges. This direction reflects growing interest in linking large-scale geodynamic processes to mineral system formation and resource distribution. However, despite its conceptual appeal, several key scientific, methodological, and interpretive issues reduce confidence in the proposed causal mechanism. Substantial revisions are required before the manuscript can be considered for publication.
Major Comments:
1. Subduction has not been constant since 1.8 Ga. Trench length, subduction flux and slab geometry changed through time. If subduction controls sediment-hosted mineralisation, these variations should influence metal endowment. However, total metal content shows no clear correlation with age or with subduction input. The authors should test this directly or explain the lack of a temporal signal, which currently weakens the proposed first-order control.
2. The manuscript assumes that craton margins at similar distances from a trench experience comparable geodynamic forcing. Yet subduction systems vary widely in slab dip, rollback rate, convergence velocity, slab age and thermal structure. These differences strongly influence mantle return flow, lithospheric strength, basin evolution and metallogenic fertility. Therefore, two margins located 1,000–1,800 km from a trench may not be tectonically equivalent. The authors should discuss how subduction style and dynamics may modify, reinforce or limit the proposed distance relationship.
3. Uncertainty is significantly higher before ~1.0 Ga, particularly in trench location and longitudinal motion. The authors should quantify uncertainty ranges or test alternative reference frames. Do not rely solely on Cao et al. (2024) to calculate the distance.
4. The manuscript appears to imply that subduction primarily perturbs and reactivates pre-enriched lithospheric reservoirs rather than directly supplying ore-forming metals or fluids. If this is the intended model, the authors should state this more explicitly and provide supporting evidence regarding metal and volatile sources.
5. The ASPECT simulations are two-dimensional and rely on imposed surface velocities, which inherently limit the ability to capture fully three-dimensional mantle flow, slab-rollback geometries, cratonic keel morphology, and associated dynamic feedbacks. These modeling constraints should be explicitly acknowledged. In addition, the modeled peak strain is located 1,500–2,500 km from subduction zones, systematically farther than the observed 800–1,800 km window. Although the authors offer a reasonable explanation, future incorporation of 3-D geodynamic models may provide a more realistic test of the proposed mechanism and potentially reconcile this offset.
6. The geodynamic models appear to use present-day cratonic lithospheric thickness and margin geometries derived from modern tomography. However, craton margins are the parts of continents most susceptible to thermal, mechanical, and metasomatic modification through time, and their lithospheric thickness likely differed substantially at 1.8 Ga, 1.2 Ga, 600 Ma, or 250 Ma. Because the efficiency and spatial reach of subduction-driven return flow depend strongly on lithospheric

thickness and viscosity structure, the choice to use modern thickness parameters requires justification. At minimum, the authors should acknowledge this assumption, explain why present-day values are appropriate, or discuss how temporal variations in margin thickness may influence the inferred distance window and the proposed mechanism.
Some Detailed Comments:
1. Several minor language and formatting issues are present throughout the manuscript, including inconsistent use of articles, hyphenation, terminology (craton edge vs craton margin), numerical formatting, and stylistic repetition of transition words. A careful editorial proofreading would improve clarity and consistency.
2. Avoid assertive language like “establish” or “prove”. In many cases, it’s better to use “suggest”, “indicate” or “support”.
3. Some figure should have scale bars or latitude/longitude.
In summary, this manuscript presents an ambitious framework and provides extensive statistical analysis and numerical calculations to support a spatial association between sediment-hosted mineralisation and craton-trench distance, which are very attractive. However, the current form does not yet convincingly demonstrate a causal linkage nor adequately address uncertainties inherent in deep-time plate reconstructions and geodynamic model simplifications. The manuscript would benefit from more rigorous treatment of these issues, refinement of over-assertive phrasing, clearer justification of methodological choices, and deeper discussion of alternative interpretations and metallogenic processes. Major revision is required before the manuscript can be considered again.

(Remarks on code availability)
It's not my expertise

Version 1:

Reviewer comments:

Reviewer #1

(Remarks to the Author)
This is my second review of this manuscript. In the previous round of review, there are several common issues raised by the reviewers. A major concern is about the uncertainty of possible dynamic relationship between the subduction-induced far-field overriding lithospheric deformation and the sediment-hosted mineralization. In the revised version, the authors have tried to adjust the details of the numerical models to strengthen this connection, by localizing the deformation beneath the craton margin. Again, I think the topic is interesting; however, the mechanism itself is not very robust.
On the one hand, the relationship between the subduction-induced “weak” strain localization at the bottom of cratonic edge is kind of dynamically far from the sediment-hosted mineralization. As argued in the abstract “this mechanically driven weakening promotes rifting, enhances permeability, and facilitates the infiltration of slab-derived volatiles, thereby preconditioning the lithosphere for metallogenesis”, however, all these processes are not modeled or analyzed in this study. Thus, this is just an inference.
On the other hand, as also raised by other reviewer(s), the subduction-induced mantle flow is strongly dependent on the slab geometry, subduction velocity, etc; then, how can the strain localization occur within a similar range of distance? I acknowledge that the authors have made arguments on this point “The length of individual trenches and slab geometry fluctuates over time, and we recognize that these changes may lead to local deviations from the global distance pattern. Our framework provides a broad, global-scale tectonic and geodynamic context, while acknowledging that variability in subduction styles can influence, shift, or limit the expression of this distance relationship in specific regions. This is why we observe that distances are not fixed but tend to cluster between 800 and 1800 km.” I am still not convinced. For example, in the present-day Earth, there are many subduction zones with contrasting geometries and velocities, does the mantle flow induced strain localization in the overriding plate agree with this inference?
Based on such kind of issues, I have to say that the uncertainties in the proposed mechanism are large, which prevent the establishment of a strong, mechanical relationship between the subduction-induced mantle flow and far-field crustal mineralization in the overriding plate.

(Remarks on code availability)

Reviewer #2

(Remarks to the Author)
Thank you for revising the manuscript and taking the time to respond to all of the comments. I would like to request some of the rationale given in R10 of the response be added to the Methods in the paragraph starting on line 381. Aside from that, I am satisfied with the revisions. Thanks.

(Remarks on code availability)

Reviewer #3

(Remarks to the Author)
The manuscript has improved substantially after revision. The authors have addressed the main concerns raised in my

previous review, clarified the scope of the study and strengthening the discussion of limitations. This study offers a useful perspective by linking cratons, typically regarded as stable and largely unaffected by subduction, to mineralisation along their margins. I recommend acceptance after minor revision.
Main comment
The authors now make it clear that the numerical models are not intended to directly simulate crustal processes, but rather to identify a first-order geodynamic control (subduction-driven mantle return flow) that may precondition craton margins for mineralisation. This clarification largely resolves my previous concerns about the link between deep lithospheric strain and shallow crustal metallogenesis. However, it should keep a clear distinction between model results in this study and inferred geological processes in the discussion.
Minor suggestions
In Figure 1, the symbol size appears to represent deposit size, but this relationship is not explained in the figure legend. Please clarify this.
In Figure 4, the lithosphere–asthenosphere boundary appears to contain green-coloured regions, whereas this colour does not appear in the corresponding colour bar. Please re-check the plotting routine and colour bar settings.
And all Figure references and captions should be checked for consistency.

Additional feedback:

First, I agree with Reviewer #1 that the proposed mechanism is not directly demonstrated and remains partly inferential. More broadly, the mechanism remains primarily supported by statistical relationships and probabilistic inference, and the process-based constraints remain somewhat uncertain. In addition, the predicted subduction-induced mantle flow likely depends on several other parameters that are not fully explored in the current framework. The treatment of subduction geometry is also quite simplified (e.g. slab angles limited to 5°–30°), whereas natural systems span a much wider range (e.g. Ficini et al., 2017). In addition, possible effects of residual slabs or inherited subduction structures beneath cratons are not considered and could be briefly acknowledged.

Some figures would benefit from closer checking. For example, Fig. 3 could be presented more clearly. There is noticeable overlap between the deposits and the random points, and the overall pattern appears rather scattered, so a more cautious interpretation would be helpful. In Fig. 4, the lithosphere–asthenosphere boundary appears to include green-coloured regions, but this colour is not shown on the corresponding colour bar, making the figure difficult to interpret. In addition, the code shared on Zenodo appears to remain at last year’s version and does not seem to include the ASPECT component. This makes it difficult for reviewers to fully assess and reproduce the modelling results.

The main issue is how the current results are interpreted. The authors should focus more on explaining why these parameters were chosen and how they relate to the geological conditions of deposit formation, rather than carrying out further modelling.

In my view, the remaining issues mainly concern the level of constraint and the way the results are presented. A clearer distinction between model results and geological interpretation, together with more cautious wording and some additional discussion, should be sufficient to address them.

(Remarks on code availability)

Version 2:

Reviewer comments:

Reviewer #3

(Remarks to the Author)
The manuscript has improved substantially after revision. The authors have made an effort to respond to the previous comments, and the scope and limitations of the study are now better defined. Linking cratons, which are often treated as stable and largely unaffected by subduction, to mineralisation along their margins is an interesting idea and worth exploring. That said, I think some aspects still need further revision before the manuscript can be accepted.

In Fig. 1, the symbol size appears to represent deposit size, but this is not explained in the legend.
Fig. 3 could be presented more clearly. There is noticeable overlap between the deposits and the random points, and the overall pattern appears rather scattered, so a more cautious interpretation in the caption would be helpful.
All figures and their captions should also be checked for consistency. In addition, the code shared on Zenodo appears to remain at last year’s version and does not include the ASPECT script file used in this manuscript. This makes it difficult for reviewers and readers to fully assess and reproduce the modelling results.

(Remarks on code availability)

Open Access This Peer Review File is licensed under a Creative Commons Attribution 4.0 International License, which permits use, sharing, adaptation, distribution and reproduction in any medium or format, as long as you give appropriate credit to the original author(s) and the source, provide a link to the Creative Commons license, and indicate if changes were made.
In cases where reviewers are anonymous, credit should be given to 'Anonymous Referee' and the source.
The images or other third party material in this Peer Review File are included in the article's Creative Commons license, unless indicated otherwise in a credit line to the material. If material is not included in the article's Creative Commons license and your intended use is not permitted by statutory regulation or exceeds the permitted use, you will need to obtain permission directly from the copyright holder.
To view a copy of this license, visit https://creativecommons.org/licenses/by/4.0/

REVIEWER COMMENTS

Reviewer #1 (Remarks to the Author):

Review of the manuscript “How Subduction Evolution Drives Sediment-Hosted Mineralisation Along Craton Edges” by Shirmard et al. submitted to nature communications. This study comprises a two-step investigation. Firstly, the authors find that the metallogenesis clusters at the craton margins that are 800–1,800 km from subduction zones, based on the global plate motion model and a global mineral deposit database. Then, several 2D geodynamic models have been conducted with ASPECT software, which demonstrate that locations of elevated stress and strain along craton edges coincide with subduction-driven mantle return flow at similar distances from subduction zones. Finally, they propose that subduction may play a first-order control on craton-margin mineralisation, based on the inference that “mantle return flow weakens craton margins, promotes rifting, channels fluids into the crust, enriching the lithosphere with volatiles, and fostering metallogenesis”.

Comments 1-1:

The paper is well-targeted and clearly illustrated. My main concern lies in the (weak) connection between simple numerical models and the complex process of metallogenesis. (1) I am not very convinced by the relationship between the modelled relatively high strain rate at the bottom of craton edge and the metallogenesis in the shallow crust. Firstly, the high strain rate is widely distributed beneath the continental plate (e.g. in Figure 5), although a peak is predicted at the craton edge by integrating vertically between 100–300 km. I am curious about the vertical integration; why is 100–300 km used. The lithosphere thickness of craton is 300 km, but that of thin continental plate is up to 150 km. Thus, if you integrate between 100–300 km, the high strain rate region beneath craton will not be included.

Response 1-1:

We thank the reviewer for this thoughtful comment. We chose 100 – 300 km to focus specifically on lithospheric and immediately sublithospheric strain under continental margins and cratons. 300km denotes the maximum thickness of the craton, and 100km is the lower bound on continental margin thickness initially. We want to avoid stress amplification in regions of high viscosity (near surface) and high asthenospheric strain rates. The cratonic thickness is generally thinned somewhat, and thus 300 km includes the rheological boundary layer, but avoids most of the asthenospheric flow. The selection criterion is not perfect, but it is the least arbitrary we could envisage. As shown in the numerical models that plot the strain field, it uniquely captures the strain features of interest.

Our intention is not to imply that the numerical models directly simulate shallow-crustal ore-forming processes or brittle strain localisation. Rather, the models are used to quantify a **far-field geodynamic driver**—namely subduction-induced mantle return flow—that preferentially focuses stress and strain at craton margins. This deep-seated strain localisation is interpreted as a first-order control that can promote lithospheric weakening, magma ascent, and fluid focusing in the overlying crust, thereby creating favourable conditions for metallogenesis.

The vertical integration range of **100–300 km** was selected to capture strain within the **mid-to lower lithosphere and the immediately underlying rheological boundary layer**, where subduction-driven return flow most effectively shears the base of the continental lithosphere. The upper bound of 300 km corresponds to the maximum thickness of cratonic lithosphere in the model, while the lower bound of 100 km approximates the initial thickness of thinner continental lithosphere at craton margins. This choice allows a consistent comparison between cratonic and non-cratonic domains.

Importantly, this window excludes (i) the shallow, high-viscosity part of the lithosphere where brittle faulting—explicitly not resolved by our continuum visco-plastic rheology—would dominate, and (ii) the deeper asthenosphere where high strain rates reflect background mantle flow rather than lithosphere–mantle coupling. Although strain is broadly distributed beneath the continental plate, vertical integration over this depth range uniquely enhances the signal associated with basal lithospheric shearing at craton edges, which is the process of interest here.

We acknowledge that any integration window involves some degree of arbitrariness; however, this range represents the most physically motivated compromise and, as demonstrated by the model strain fields, effectively captures the key strain localisation features relevant to the proposed geodynamic–metallogenic link.

In Response 1-2, we explain how we link sublithospheric strain focusing to upper-crustal deformation and metallogenesis.

Revision to Discussion:

Strain rates are vertically integrated between 100–300 km to isolate deformation within the mid- to lower lithosphere and the immediately underlying rheological boundary layer, where subduction-driven mantle return flow most effectively shears the base of the continental lithosphere. The upper bound of 300 km corresponds to the maximum cratonic thickness in the model and includes the basal portion of the cratonic root, which is commonly somewhat thinned in dynamic settings. The lower bound of 100 km approximates the initial thickness of adjacent thinner continental lithosphere. This depth window avoids stress amplification in the shallow, high-viscosity lithosphere—where brittle processes are not explicitly resolved in our rheology—as well as the higher background strain rates of the deeper asthenosphere that are not directly related to lithosphere–mantle coupling. Although elevated strain is distributed more broadly beneath the continental plate, integration over this interval preferentially highlights strain localisation at craton margins. While the selection is not unique, it represents a physically motivated and internally consistent criterion that captures the strain features most relevant to lithospheric weakening at craton edges.

Comments 1-2:

Secondly, I am wondering how to connect the relatively high strain rate beneath the continental lithosphere (induced by mantle flow shearing) to the shallow crustal deformation and metallogenesis. It is inferred that “mantle return flow weakens craton margins, promotes rifting, channels fluids into the crust, enriching the lithosphere with volatiles, and fostering metallogenesis”. However, all these processes are not predicted in the present numerical models; the continental lithosphere in these models is kind of rigid without any strain localization (Figures 5, and S1–S4).

Response 1-2:

Thank you for your valuable comment. We cannot model all processes in a single numerical framework and therefore intentionally avoid explicitly simulating continental deformation. The rationale is that the effect of interest demonstrably occurs within stable continental terranes, where large-magnitude surface deformation is not required. Our numerical models instead resolve significant strain in the lower lithosphere, sublithosphere, and asthenosphere, which we interpret as being associated with smaller-scale, sub-resolution strain localisation within the continental lithosphere and crust.

While shallow crustal deformation, rifting, fluid flow, and metallogenesis are not directly predicted by our models, previous lithospheric-scale studies show that sustained basal shearing and mantle-driven forcing can be mechanically coupled to the upper lithosphere over geological timescales (e.g. McKenzie, 1978; White, 1993; Newman & White, 1997). In natural systems, craton margins are characterised by inherited rheological weaknesses such as ancient shear zones and rifted margins, which efficiently localise strain and channel fluids even under modest far-field stresses.

Accordingly, our models are intended to capture a first-order geodynamic control—subduction-driven mantle return flow—that spatially focuses lithospheric weakening at craton edges. These conditions are widely recognised as prerequisites for basin formation, fluid circulation, and sediment-hosted mineral systems, as established by existing ore-system models. We will clarify in the revised Discussion that our approach provides a geodynamic framework that complements, rather than explicitly simulates, crustal deformation and metallogenesis.

Revision to Discussion:

Our numerical models do not explicitly resolve continental deformation, fluid flow, or ore-forming processes. Instead, they isolate a first-order lithospheric-scale forcing mechanism that operates even within stable continental terranes. The models predict enhanced strain in the lower lithosphere and underlying mantle associated with subduction-driven return flow. We interpret this deep-seated strain as being mechanically coupled, over geological timescales, to smaller-scale and sub-resolution deformation within the continental lithosphere and upper crust, particularly along inherited craton-margin structures. These conditions are widely recognised as prerequisites for basin development and fluid circulation in sediment-hosted mineral systems. Accordingly, our models provide a geodynamic framework that complements, rather than directly simulates, crustal deformation and metallogenesis.

Comments 2:

(2) The observed deposit distance from subduction trench is 800–1800 km, whereas the numerically modelled strain maxima beneath the continent is 1500–2500 km. The authors argue that they are slight offset and attribute this discrepancy to the possible inherited lithospheric heterogeneities and 3D mantle flow patterns that are not considered in the present model. In my opinion, this offset is not small enough to be neglected. I would suggest improving the model, e.g. including the factors as proposed, to find the controlling factor for such a trend of metallogenesis.

Response

We thank the reviewer for this very constructive comment. Following this suggestion, we carefully re-examined our numerical models and distance calculations, and have run additional models. The mismatch in spatial scaling across the five models has now been alleviated. After adding additional models and correcting the distance calculations, the agreement between observed deposit distances and modelled strain maxima improves substantially. The revised results show a good correlation with an offset of ~100 km in their peaks, which we consider reasonable given model simplifications, uncertainties in plate reconstructions, and the absence of fully three-dimensional mantle flow and inherited lithospheric heterogeneity in the models. We have updated the figures and text accordingly and clarified these points in the revised manuscript.

Comments 3:

(3) By the way, the subducting slab-induced mantle flow pattern is strongly controlled by the length and angle of sinking slab. In this sense, it is time-dependent. In this study, a constant model time of 27 Ma is used. Although it is argued that it reaches the steady-state, I am kind of curious about that, since the slab continues sinking and deforming. I would suggest plotting some time-dependent snapshots to check whether the high strain rate always localizes beneath the craton edge.

As a summary, I agree that the story is big and attractive; however, it requires many improvements to build the 'solid' connection between subduction-induced mantle flow and the crustal-scale metallogenesis, rather than a bunch of inferences.

Response 3:

We thank the reviewer for raising this important point. Although we report results at 27 Ma, we confirm that the deformation pattern is **not an artefact of this single timestep**. In our models, the slab reaches a quasi–steady-state geometry after ~20–25 Ma, after which the return-flow cell and the location of maximum strain beneath the overriding plate become **stable in space**. We have now added **time-dependent snapshots** (new Figure Sx) showing that the craton-edge strain maximum persists from ~20 Ma to 40 Ma, despite ongoing slab sinking and thickening. This demonstrates that the localisation is a robust consequence of the large-scale return-flow geometry rather than of model timing.

We agree that our study focuses on first-order geodynamic forcing and does not explicitly model crustal metallogenic processes. We now clarify these limitations and more carefully frame the inferred links as **mechanistic connections supported by existing mineral-systems literature**, while acknowledging that more detailed 3D and time-dependent models will be needed to fully resolve the lithosphere-to-crust pathways.

We have plotted a timeseries in the Supplementary data, demonstrating that the cratonic strain (and stress) remains localized around the boundary over the evolution of a subduction zone, from incipient subduction to slab breakoff.

Comments 4:

Some minor points:

C4-1: ---The "MATERIALS AND METHODS" is organized rather loosely, for example, with many short sections and repeated contents.

R4-1: Thank you for your comment. We have reorganized the Materials and Methods section to reduce repetition and improve the overall structure.

C4-2: ---Lines 317-318, I do not think Fig. 3 and Fig. 4 are correctly cited.

R4-2: We thank the reviewer for noting this. We have corrected the figure citations.

C4-3: ---Lines 401-408, I do not know why the initial model configuration appears here, instead of in the section of "Numerical geodynamic model".

R4-3: We agree that this material should appear in the "Numerical Geodynamic Model" section. We have moved the description of the initial model configuration to that section for clarity and improved structure.

Reviewer #2 (Remarks to the Author):

In this study, the authors explore the potential geodynamic reasons behind the observation that major sediment-hosted base metal deposits are found on some (but not all) craton margins. They build their own database of craton margins and use a plate reconstruction to demonstrate that mineralised margins are likely to occur when there is a distance of 800-1800 km between that margin and a subduction zone. They then use 2-D geodynamic models of subduction zones to demonstrate that craton margins become sites of strain localisation at roughly this distance from trenches. They use these combined pieces of information to suggest that localised weakening of the sub-continental lithosphere at these locations provides optimal conditions for metasomatism, providing a means of boosting their fertility and preconditioning the location for mineral deposit formation.

I enjoyed reading your study and think it adds some interesting additional insights into this widely known observation. In my opinion, this material is worthy of publication in a journal with the stature of Nature Communications. Nevertheless, I have a number of points that need to be addressed before I would approve it for publication.

General points:

Comment 5:

Enhanced predictability: The premise of this study is to improve our ability to predict deposit locations and this point is central to your conclusions. But where is the predictability? There doesn't seem to be a map of which of the present-day boundaries should be more or less prospective based on your results. But this aspect is likely to be the study's most useful output if the knowledge is going to be translated into exploration workflows.

Response 5:

We appreciate the reviewer's point regarding predictability. Our primary aim in this study is to establish the **first-order geodynamic control**—the characteristic trench-to-craton distance window—rather than to produce a full mineral prospectivity map. We now clarify in the revised text that this work is intended to **identify the governing physical mechanism** linking subduction-driven mantle flow to craton-margin mineralization. Translating this insight into **predictive, spatially explicit prospectivity maps** is our long-term plan and requires the incorporation of additional datasets (e.g., basin architecture, structural inheritance, magmatic history, along with many other plate tectonic features), and we note that this represents a **separate, future project** beyond the scope of the present study. We have updated the conclusion to emphasise this distinction and to state that the framework developed here can serve as a foundation for future predictive mapping.

Comment 6:

Craton margins: You have used an AI-based approach to map the craton boundaries that are essential to your study, but have provided very scant details on the procedure and how well the model has performed. For starters, I do not think that I could remotely repeat this process. How does it work? What challenges did you need to think about to make it work? Is it possible that these are the same ones from your Economic Geology paper? If so you should certainly cite it!

Secondly, how well has the algorithm done? There's no real comparison with other models and you should at least repeat the CDF of deposits versus random locations in the continents for this present-day setup to demonstrate that these boundaries are as good as (or better) than previous models. There is one brief mention in the Figure 8 caption of an SGTSG abstract, but that is insufficient evidence for a reader to pass judgement on these boundaries.

Response 6:

Thank you for your comment. We apologise—We mistakenly cited the conference paper instead of the newly published paper in *Geoscience Frontiers as below:*

(https://doi.org/10.1016/j.gsf.2025.102176).

We have corrected the citation, and all relevant details are now available in the updated paper. In this version, we used published thermal and lithospheric thickness models, and distinguished cratons from other thick lithospheric features and identified their edges and associated deposits. We are inspired by the Hoggard et al. (2020) boundaries and improved the boundaries by reducing the search space for sediment-hosted, craton-related deposits from 200 km to 90 km, thereby improving the detection of craton margins at the global scale.

Comment 7:

Deposits: I'm a bit concerned about your deposit databases. The ones you show in Figure 1 (top-left panel) and Figure 8 are not consistent with one another. And both are missing some major deposits that are in the databases of Hoggard et al., which is stated as where this database is taken from. For example, none of the major deposits in the Middle East show up. It is important that you explain these differences to avoid being accused of cherry-picking data. I personally don't like any removal of deposits from the original database if you are then generating CDFs and making statements about doing better than the original Hoggard et al. (2020) study. That would not be a fair comparison and you would need to perform the same analysis on the Hoggard et al. LAB maps.

Response 7:

Thank you for pointing this out. The inconsistency between the deposits shown in Figure 1 (top-left panel) and Figure 8 occurred because we forgot to filter out deposits without age information in Figure 8. This has now been corrected.

Unlike Hoggard et al. (2020), our study separates craton-related deposits from other thick-lithosphere-margin deposits. The craton-related category encompasses more than 85% of the deposits (total metal content) included in Hoggard et al.'s database. All details of how this separation was performed—using a decision-tree approach—are fully explained in the published paper in Geoscience Frontiers (https://doi.org/10.1016/j.gsf.2025.102176).

Revision to the Materials and Methods section based on comments 6 and 7.

We separated craton-related deposits from other thick-lithosphere-margin deposits to focus specifically on craton-associated ore bodies. This distinction was made using a decision-tree approach, which allowed us to identify and isolate craton-related deposits, encompassing over 85% of the total metal content from the original database. We chose this approach to better capture the relationship between craton margins and metallogenesis.

The boundaries of these cratons improved the previous work at detecting craton boundaries[33], reducing the search space for craton-related deposits from 200 km to 90 km, resulting in more accurate craton edge identification. (*https://doi.org/10.1016/j.gsf.2025.102176*).

Comment 8:

Language: The manuscript can sometimes be quite repetitive and jumps back and forth. For example, you start the Geodynamic Model Results section with a sentence stating the results (lines 164-165), but this is then repeated on lines 189-191. You also have a statement in lines 169-170 about reasons for differences between your models and observations, but then different reasons are given in lines 220-223. Lines 229-235 seem to potentially say the same thing twice. The key ligands listed are different in Lines 218 and 241. The Materials and Methods section covers the craton boundaries and deposit databases in two places. The numerical modelling also seems to be split into two sections in different places. Apologies if I'm missing something, but this section needs to be appropriately streamlined and the rest of the text tidied up.

Response 8: Thank you for the feedback. We reviewed the manuscript to remove repetitions, ensure consistency in reported results and key details, and streamline the Materials and Methods and Results sections for clarity and logical flow.

Comment 9:

Specific points:

C1-Introduction: Your study's motivation and methods seem to be heavily inspired by the study of Hoggard et al., (2020). It might be good to actually cite it somewhere in the main text? Perhaps in the introduction?

R1-Thank you for the suggestion. We have now incorporated a citation to Hoggard et al. (2020) in the second sentence of the introduction as well as in the materials and methods.

C2-Figure 1: If your causal mechanism is about slabs fertilising the lithosphere prior to deposit genesis, why not show the deposit locations at periods prior to their formation time? These panels are also very small! And the time labels are not clearly associated with their respective panels (e.g. they are closer to the panel above).

R2- We thank the reviewer for this helpful suggestion. In response, we have revised Figure 1 and added an additional figure and increased the size of all plots to improve readability and clarifying the visual association between time and each snapshot. To avoid ambiguity, deposit names have been removed, and instead the deposits formed at those time slices are clearly labeled using large fonts, with red rectangles highlighting their positions relative to their subduction zones.

We considered showing deposit locations at times prior to their formation; however, doing so would have required at least doubling the number of panels, which significantly reduced clarity and made the figure difficult to interpret. Our revised approach focuses on the configuration at the time of deposit formation, which directly addresses the relationship between deposit location and proximity to subduction.

To ensure that the temporal evolution of subduction and plate configurations remains accessible, we explicitly refer readers to the accompanying movie, which illustrates the

progressive development of subduction systems through time and provides the full temporal context for deposit formation.

C3-Figure 3: This figure is interesting! But the caption is very brief and uninformative. Also what are the units of the density contours? I'm also not sure about your conclusion that deposits are more likely to be on cratons undergoing trench advance compared to other locations – whilst they do have negative trench-normal motion, it is less extreme than for the random locations (based on the density contours). Some might therefore argue that you want a less strong trench advance?

R3-Thank you for your feedback! We have revised both the paragraph and the figure caption for Figure 3 to clarify the interpretation. Specifically, we've expanded the caption to explain that the density contours represent a normalized probability distribution, with no physical units. Additionally, we emphasized that deposits are predominantly located in the **negative trench-normal motion** zone (trench retreat), despite random points being distributed in both the positive and negative zones. We also refined the discussion to suggest that **moderate trench retreat**, rather than extreme trench retreat or advance, likely provides the optimal conditions for deposit formation.

C4-Figure 4: Sorry, but this figure is not so good. It is hard to interpret the information, there is no key for the colours, and much of the information has already been shown in Figure 3. Can you perhaps make the three deposit classes into three different shaped symbols in Figure 3 and then colour them by age? Then you could drop this Figure 4.

R4- We thank the reviewer for this helpful suggestion. We have removed the original Figure 4 from the main text and moved it to the supplementary materials. While we appreciate the idea of integrating deposit classes as different symbol shapes in Figure 3 and colouring them based on their age, we prefer to retain the current design to maintain consistency across all figures and the accompanying movie. However, we have revised the caption of Figure 3 to provide greater clarity.

C5-Lines 168-170. None of these reasons seem to explain a systematic under-prediction of the distance?

R5- We thank the reviewer for this helpful comment. As explained in detail in our responses above, we have revised the model setup and distance calculations, which were the primary source of the apparent systematic under-prediction. After correcting the model scaling and measuring distances at the time of deposit formation rather than at subduction initiation, the offset is reduced to ~100 km. At this scale, the residual difference is within the uncertainties of plate reconstructions and model simplifications, and no longer represents a systematic under-prediction. We have updated Lines accordingly to reflect this revision.

C6-Figure 5: This figure is important. Can you make the flow arrows more visible (they are too faint). Also don't put the two scale bars on top of the domain. The y-axis labels on the upper panel need appropriate formatting.

R6- We thank the reviewer for highlighting the importance of this figure. The flow arrows have been enhanced to improve their visibility, the scale bars have been repositioned outside the

model domain, and the y-axis labels in the upper panel have been reformatted for clarity. The figure has been revised accordingly.

C7-Figure 6: Why not plot this result for all five of the individual models? There are also typos in the axis labels and the units need formatting.

R7-We appreciate the reviewer's comment and understand how the misunderstanding arose. The current plot already presents the results for all five individual models. To avoid this misconception, we have revised the figure caption and removed the term "profile," which may have implied otherwise. We have also corrected the typos in the axis labels and improved the formatting of the units. We have also superimposed the strain rate graph onto the plot showing the histogram of distances between craton margins and the nearest subduction trench.

C8-Line 316: Referencing error.

R8-Thank you for pointing this out. We have reviewed the reference, although it did not show any problem in the doc file, but it was removed and re-added to make sure it is correct.

C9-Line 329: Here you refer to IOCG deposits, but I don't see them elsewhere in the study?

R9-Thank you for your comment. We have revised the corresponding sentence and removed the reference to IOCG deposits, as they were not part of the focus of this study.

C10-Line 361: What is the rationale for driving these 2-D synthetic subduction models with real present-day plate velocities from GPlates? Where are they derived from? Does that mean the trench is not the only site of convergence/divergence?

R10-Thank you for your comment. We use a present-day plate velocity GPML file distributed with ASPECT. It is based on the EarthByte global rotation model for the present day only. The relevant reference is: Zahirovic, S., Eleish, A., Doss, S., Pall, J., Cannon, J., Pistone, M., and Fox, P., 2022, *Subduction kinematics and carbonate platform interactions*, Geoscience Data Journal, doi:10.1002/gdj3.146.

The simulation represents a 2-D spherical annulus extracted along the equator from that global plate model, and thus multiple sites of convergence and divergence are present within the computational domain. This produces a more realistic large-scale mantle flow geometry than an artificially prescribed velocity field (although, in the revised manuscript, we also include a simplified configuration for comparison). The selected subduction zone is the most stable subduction locale within the annulus, ensuring that the trench–craton distance remains consistent over the lifetime of the simulation.

The rationale for using present-day GPlates-derived velocities is to ground the synthetic models in an internally consistent, observation-based plate kinematic framework rather than prescribing arbitrary convergence rates. While multiple plate boundaries exist in the annulus, our analysis focuses exclusively on the chosen subduction zone as the primary site of convergence relevant to this study. Other boundaries are present as part of the global kinematic system but are not the focus of interpretation.

C11-Table 1: Where have these numbers come from or what is the justification for choosing them?

R11: We value your careful consideration and feedback. We have added additional auxiliary references in the supplementary, with justifications for the parameters chosen.

C12-Figure 7: Thank you – this is a helpful figure for summarising your workflow.

R12- We are so glad to see your kind words!

C13-Figure 8: Given these craton boundaries are a central component of your study, you should justify why you have made them as opposed to using previously published ones. This links back to my previous comment about explaining where these new ones come from. You need to demonstrate that your workflow is believable and does provide a good present-day match to the deposits (in my opinion, including those currently missing from this figure!). I think that this figure should also be in the main text (probably as Figure 1?).

R13-Thank you for your comment. As mentioned in a previous response, we mistakenly cited the conference paper instead of the newly published paper. The correct reference is now provided below, with full details and answers regarding your questions available in our *Geoscience Frontiers* publication (https://doi.org/10.1016/j.gsf.2025.102176). Our approach builds upon and improves the craton boundaries from Hoggard et al. (2020) by refining the search space for craton-related deposits, which has led to a more accurate detection of craton margins on a global scale. We employed a decision-tree method to distinguish cratons from other thick-lithosphere features.

Reviewer #3 (Remarks to the Author):

This manuscript addresses an important and actively debated question in economic geology: why sediment-hosted metal deposits preferentially develop along craton margins. The integration of global plate reconstructions, seismic-defined craton boundaries, metal deposit databases, statistical analyses and numerical geodynamic simulations is ambitious and has the potential to generate meaningful insights. Shirmard et al. (2025) proposes a unifying geodynamic framework in which subduction-driven mantle return flow promotes lithospheric weakening and enhances metallogenic fertility along craton edges. This direction reflects growing interest in linking large-scale geodynamic processes to mineral system formation and resource distribution. However, despite its conceptual appeal, several key scientific, methodological, and interpretive issues reduce confidence in the proposed causal mechanism. Substantial revisions are required before the manuscript can be considered for publication.

Major Comments:

Comment 10:

Subduction has not been constant since 1.8 Ga. Trench length, subduction flux and slab geometry changed through time. If subduction controls sediment-hosted mineralisation, these variations should influence metal endowment. However, total metal content shows no clear correlation with age or with subduction input. The authors should test this directly or explain the lack of a temporal signal, which currently weakens the proposed first-order control.

Response10:

Thank you for your insightful comment. Based on Figure 1B from the latest publication,

https://doi.org/10.1016/j.epsl.2025.119683

subduction rates have remained relatively stable, averaging around 5-7 cm/year since 1.8 Ga. While individual trench length fluctuates, and slab geometry can evolve over time, we acknowledge the limited data on these variations. We recognize that these changes may lead to local deviations from the global distance pattern. Our framework provides a broad, global-scale geodynamic context, while also acknowledging that variability in subduction styles can influence, shift, or limit the expression of this distance relationship in specific regions. This is why we observe that distances are not fixed but tend to cluster between 800 and 1800 km.

We added this paragraph into the Discussion Section: Subduction and Metal Endowment:

Additionally, subduction rates have remained relatively stable, averaging around 5-7 cm/year since 1.8 Ga. The length of individual trenches and slab geometry fluctuates over time, and we recognize that these changes may lead to local deviations from the global distance pattern. Our framework provides a broad, global-scale tectonic and geodynamic context, while acknowledging that variability in subduction styles can influence, shift, or limit the expression of this distance relationship in specific regions. This is why we observe that distances are not fixed but tend to cluster between 800 and 1800 km.

Comment 11:

The manuscript assumes that craton margins at similar distances from a trench experience comparable geodynamic forcing. Yet subduction systems vary widely in slab dip, rollback rate, convergence velocity, slab age and thermal structure. These differences strongly influence mantle return flow, lithospheric strength, basin evolution and metallogenic fertility. Therefore, two margins located 1,000–1,800 km from a trench may not be tectonically equivalent. The authors should discuss how subduction style and dynamics may modify, reinforce or limit the proposed distance relationship.

Response 11:

We thank the reviewer for this important observation. We agree that subduction systems differ in slab dip, rollback rate, convergence velocity, slab age, and thermal structure, and that these factors can modify mantle return flow and lithospheric deformation. Our distance relationship is intended as a **first-order spatial tendency**, not a prediction that all craton margins at the same trench distance experience identical forcing. We have now added a concise discussion noting that different subduction styles can (i) shift or sharpen the location of maximum strain, (ii) enhance or suppress lithospheric weakening, and (iii) influence basin formation and metallogenic fertility. The revised text emphasises that the **1,000–1,800 km distance window is an emergent global pattern**, but local deviations are expected due to subduction-style variability.

We added this paragraph to the Discussion Section to acknowledge these details:

“The trench–craton distance window identified here represents a first-order spatial tendency, not a universal condition. Variations in subduction style—including slab dip, rollback rate, convergence velocity, slab age, and thermal structure—can alter mantle return-flow geometry and the magnitude or position of lithospheric strain. Consequently, two craton margins located at similar distances from a trench may experience different tectonic forcing. These differences likely contribute to local departures from the global distance pattern. Our framework therefore

provides a broad geodynamic context, while recognising that subduction-style variability can reinforce, shift, or limit the expression of the distance relationship in specific regions.”

Comment12:

Uncertainty is significantly higher before ~1.0 Ga, particularly in trench location and longitudinal motion. The authors should quantify uncertainty ranges or test alternative reference frames. Do not rely solely on Cao et al. (2024) to calculate the distance.

Response12:

We agree that uncertainty increases substantially before ~1.0 Ga and appreciate the reviewer’s concern. Because **Cao et al. (2024) is currently the only global, continuously evolving reconstruction from the present day to 1.8 Ga**, no alternative full-planet reference frames exist for direct comparison.

Comment13:

The manuscript appears to imply that subduction primarily perturbs and reactivates pre-enriched lithospheric reservoirs rather than directly supplying ore-forming metals or fluids. If this is the intended model, the authors should state this more explicitly and provide supporting evidence regarding metal and volatile sources.

Response13:

Thank you for your comment. We revised the Discussion section (subsection on “Subduction Zones and Craton-Margin Metallogenesis”) as below:

We propose that subduction primarily perturbs and reactivates pre-enriched lithospheric reservoirs rather than directly supplying ore-forming metals or fluids. This is evident from our geodynamic models, which indicate that subduction-driven mantle flow weakens craton margins, promoting rifting and channeling fluids into the crust. These fluids, enriched with volatiles such as sulfur, carbon, and water (ref), contribute to the metallogenesis of sediment-hosted deposits. As such, we do not suggest that subduction directly supplies metals to the mineralising systems, but rather that it creates the necessary conditions for metal transport and precipitation by enriching the mantle and lithosphere with volatile components. This process aligns with previous studies that highlight the role of mantle metasomatism in the formation of ore systems.

Comment14:

The ASPECT simulations are two-dimensional and rely on imposed surface velocities, which inherently limit the ability to capture fully three-dimensional mantle flow, slab-rollback geometries, cratonic keel morphology, and associated dynamic feedbacks. These modeling constraints should be explicitly acknowledged. In addition, the modeled peak strain is located 1,500–2,500 km from subduction zones, systematically farther than the observed 800–1,800 km window. Although the authors offer a reasonable explanation, future incorporation of 3-D geodynamic models may provide a more realistic test of the proposed mechanism and potentially reconcile this offset.

Response14: We thank the reviewer for this constructive comment. We explicitly acknowledge that the ASPECT simulations are two-dimensional and rely on imposed surface velocities, which limits their ability to capture fully three-dimensional mantle flow, slab rollback, complex cratonic keel geometries, and associated dynamic feedbacks. These modelling constraints are now clearly stated in the revised manuscript.

Regarding the apparent offset between modelled strain maxima and observed deposit distances, as explained in detail in our responses above, we have revised the model setup and distance calculations, which were the primary source of the previously inferred systematic bias. Specifically, distances are now measured at the time of deposit formation rather than at subduction initiation, and inconsistencies in model scaling have been corrected. Following these revisions, the offset is reduced to ~100 km. At this scale, the residual difference falls within uncertainties related to plate reconstructions, simplified rheology, and the two-dimensional model geometry, and no longer represents a systematic under-prediction. We have updated Lines 168–170 accordingly.

We agree that future three-dimensional geodynamic models incorporating slab rollback and realistic cratonic root geometries will provide an important avenue for further testing and refinement of the proposed mechanism, and we now explicitly highlight this as a direction for future work.

"We impose plate velocities here to both control the experimental configuration and also to explore sublithospheric flow for realistic plate motion. However, this configuration limits our ability to fully explore slab rollback, break-off, and other three-dimensional effects, including the three-dimensional architecture of cratonic roots. As a result, we focus on the asthenospheric return flow around craton margins in response to subduction, to which these models are well suited."

We have run additional subduction models under controlled subduction conditions, to both understand the sensitivity in distance and factors affecting it, and demonstrate the return flow effect on cratonic margin strain under controlled conditions. We have included the following in our Supplementary material:

Comment15:

The geodynamic models appear to use present-day cratonic lithospheric thickness and margin geometries derived from modern tomography. However, craton margins are the parts of continents most susceptible to thermal, mechanical, and metasomatic modification through time, and their lithospheric thickness likely differed substantially at 1.8 Ga, 1.2 Ga, 600 Ma, or 250 Ma. Because the efficiency and spatial reach of subduction-driven return flow depend strongly on lithospheric thickness and viscosity structure, the choice to use modern thickness parameters requires justification. At minimum, the authors should acknowledge this assumption, explain why present-day values are appropriate, or discuss how temporal variations in margin thickness may influence the inferred distance window and the proposed mechanism.
Some Detailed Comments:

Response15:

We thank the reviewer for this important observation. We have added a paragraph in the Supplementary section that goes over the sources of these values and their justification.

We also inserted the sentence below into Methods to clarify that the models are designed as simplified physical experiments, not paleogeometric reconstructions of craton margins.

"Our geodynamic simulations are designed as simplified physical experiments that capture the first-order interaction between subduction-driven return flow and a strong cratonic lithosphere; they are not intended to reproduce detailed paleo-geometries, time-specific lithospheric thicknesses, or the evolving configuration of individual craton margins. This approach is appropriate because cratonic interiors and many craton margins have remained relatively stable and long-lived through time—as evidenced by the preservation of major sediment-hosted deposits in these settings—despite local modification or reworking in some regions."

Comment16:

C16-1: Several minor language and formatting issues are present throughout the manuscript, including inconsistent use of articles, hyphenation, terminology (craton edge vs craton margin), numerical formatting, and stylistic repetition of transition words. A careful editorial proofreading would improve clarity and consistency.

R16-1: Thank you for your comment. We have carefully proofread the manuscript and corrected language, formatting, and terminology inconsistencies to improve clarity and consistency.

C16-2. Avoid assertive language like "establish" or "prove". In many cases, it's better to use "suggest", "indicate" or "support".

Thank you so much. We revised the manuscript accordingly.

C16-3. Some figure should have scale bars or latitude/longitude.

R16-3: Thank you so much. We regenerated the location map (Figure 7) and added scale bars and coordinate grids.

In summary, this manuscript presents an ambitious framework and provides extensive statistical analysis and numerical calculations to support a spatial association between sediment-hosted mineralisation and craton-trench distance, which are very attractive. However, the current form does not yet convincingly demonstrate a causal linkage nor adequately address uncertainties inherent in deep-time plate reconstructions and geodynamic model simplifications. The manuscript would benefit from more rigorous treatment of these issues, refinement of over-assertive phrasing, clearer justification of methodological choices, and deeper discussion of alternative interpretations and metallogenic processes. Major revision is required before the manuscript can be considered again.

Thank you for this comprehensive assessment. We addressed these concerns by re-running geodynamic modelling, strengthening the discussion of uncertainties, moderating over-assertive language, clarifying methodological choices, and expanding consideration of alternative interpretations and metallogenic processes. We believe these revisions will substantially improve the robustness and clarity of the manuscript.

Reviewer #3 (Remarks on code availability):

It's not my expertise

REVIEWER COMMENTS

Reviewer #1 (Remarks to the Author):

This is my second review of this manuscript. In the previous round of review, there are several common issues raised by the reviewers. A major concern is about the uncertainty of possible dynamic relationship between the subduction-induced far-field overriding lithospheric deformation and the sediment-hosted mineralization. In the revised version, the authors have tried to adjust the details of the numerical models to strengthen this connection, by localizing the deformation beneath the craton margin. Again, I think the topic is interesting; however, the mechanism itself is not very robust.

C1: On the one hand, the relationship between the subduction-induced "weak" strain localization at the bottom of cratonic edge is kind of dynamically far from the sediment-hosted mineralization. As argued in the abstract "this mechanically driven weakening promotes rifting, enhances permeability, and facilitates the infiltration of slab-derived volatiles, thereby preconditioning the lithosphere for metallogenesis", however, all these processes are not modeled or analyzed in this study. Thus, this is just an inference.

On the other hand, as also raised by other reviewer(s), the subduction-induced mantle flow is strongly dependent on the slab geometry, subduction velocity, etc; then, how can the strain localization occur within a similar range of distance? I acknowledge that the authors have made arguments on this point "The length of individual trenches and slab geometry fluctuates over time, and we recognize that these changes may lead to local deviations from the global distance pattern. Our framework provides a broad, global-scale tectonic and geodynamic context, while acknowledging that variability in subduction styles can influence, shift, or limit the expression of this distance relationship in specific regions. This is why we observe that distances are not fixed but tend to cluster between 800 and 1800 km." I am still not convinced. For example, in the present-day Earth, there are many subduction zones with contrasting geometries and velocities, does the mantle flow induced strain localization in the overriding plate agree with this inference?
Based on such kind of issues, I have to say that the uncertainties in the proposed mechanism are large, which prevent the establishment of a strong, mechanical relationship between the subduction-induced mantle flow and far-field crustal mineralization in the overriding plate.

R1: Responding to Reviewer Concerns on Mechanism and Distance Robustness

We thank the reviewer for this careful and constructive assessment. We agree that the proposed link between deep lithospheric strain localisation and shallow crustal mineralisation remains, at this stage, a first-order geodynamic inference rather than a directly modelled ore-forming process. We have therefore revised the manuscript to make that distinction more explicit and to moderate the wording accordingly.

Our central aim is not to model basin evolution, permeability development, volatile transport, or ore deposition directly. Rather, the purpose of the numerical experiments is to test whether subduction-driven mantle return flow can plausibly transmit stress across the overriding plate and focus strain at craton margins over distances comparable to those observed in the global deposit dataset. In that sense, the study establishes a mechanical framework for lithospheric preconditioning; the downstream consequences for rifting, permeability enhancement, and

volatile ingress are interpreted as geologically reasonable, but still indirect, consequences of sustained strain localisation.

To address this point more clearly, we now state explicitly in the Discussion that rifting, permeability enhancement, and slab-derived volatile infiltration are not direct outputs of the models, but interpretive inferences supported by the combination of: (i) the statistical clustering of deposits 800–1,800 km from trenches relative to random points; and (ii) the numerical experiments, which generate strain-rate maxima at comparable distances. We also identify the coupling of geodynamic forcing with hydrological and geochemical processes as an important direction for future work.

With respect to the robustness of the distance relationship, we emphasise that the manuscript does not propose a fixed or universal trench-to-deposit distance. Instead, it identifies a statistical distance window within which deposits preferentially occur at the global scale. More than 90% of the total metal endowment lies within 2,200 km of trenches, and most deposits cluster between ~800 and 1,800 km. The numerical models are intended to test whether this range is physically plausible, not whether every individual subduction margin should reproduce the same distance exactly.

We agree that natural mantle flow and strain localisation depend on slab geometry, convergence rate, trench length, rollback history, and lithospheric inheritance. We now acknowledge these dependencies more explicitly. At the same time, our experiments show that, across a range of trench–craton separations (~700–2,800 km), subduction-driven return flow consistently produces broad lithospheric-scale deformation with peak strain focusing near the craton edge when that edge lies within approximately 1,000–2,000 km of the trench. This result supports a preferred first-order scale of stress transmission, while still allowing for substantial regional deviation in real systems.

We have also clarified that the use of present-day plate velocities serves only to ground the models in realistic kinematics. The experiments are synthetic and are not intended as case-by-case reconstructions of individual modern subduction zones. We therefore do not claim that every present-day margin has been shown to match the inferred distance window; rather, we suggest that testing specific margins with fully 3-D, time-dependent models is a logical next step.

In response to the reviewer's broader concern about uncertainty, we have revised the manuscript to frame the mechanical link more carefully. Specifically, we now present the results as evidence for a first-order tectonic envelope that increases the likelihood of fertile craton margins, rather than as a deterministic predictor of mineralisation. We also emphasise that the strength of the interpretation lies in the convergence of three independent lines of evidence: the statistically significant spatial bias of deposits toward subduction-proximal craton edges relative to random edge points; the physically motivated return-flow mechanism that reproduces strain maxima at comparable distances; and the independent geological and geochemical evidence that subduction-related metasomatism and volatile fluxes can enhance lithospheric fertility.

To reflect these points, we have added the following text to the Discussion:

Although our models do not simulate rifting, permeability evolution, or fluid–rock interaction, they establish a first-order mechanical link between subduction-driven return flow and lithospheric weakening at craton edges. Within this framework, the promotion of rifting, enhancement of permeability, and facilitation of slab-derived volatile infiltration are inferred consequences of sustained strain localisation acting over geological timescales, consistent

with independent geological and geochemical evidence for basin development and metasomatic enrichment along craton margins.

The preferred 800–1,800 km distance range therefore represents a statistical window emerging from global data, not a fixed universal value for individual margins. Our numerical experiments show that, across a range of trench–craton configurations, subduction consistently generates broad return-flow cells with strain-rate maxima at ~1,300 km from the trench. This suggests that, despite variations in slab geometry and convergence rate, the underlying scale of subduction-driven return flow provides a robust first-order control on where deformation – and hence potential metallogenic preconditioning – is most efficiently focused

Uncertainties, Limitations, Future Work

While our framework links subduction, mantle return flow, and craton-edge mineralisation at a global, first-order level, significant uncertainties remain at the scale of individual systems. Three-dimensional slab geometry, time-dependent rollback, and local lithospheric heterogeneity can shift or suppress strain localisation relative to the idealised distance window used here. Consequently, our results should be viewed as defining a tectonic envelope that enhances the probability of fertile craton edges, rather than a deterministic predictor for every present-day subduction margin.

Reviewer #2 (Remarks to the Author):

C1: Thank you for revising the manuscript and taking the time to respond to all of the comments. I would like to request some of the rationale given in R10 of the response be added to the Methods in the paragraph starting on line 381. Aside from that, I am satisfied with the revisions. Thanks.

R1: Response:

Thank you for this helpful suggestion. We have incorporated the rationale referred to in our earlier response into the Methods paragraph beginning at line 381, so that the basis for the parameter choice is now explained directly in the manuscript.

Reviewer #3 (Remarks to the Author):

The manuscript has improved substantially after revision. The authors have addressed the main concerns raised in my previous review, clarified the scope of the study and strengthening the discussion of limitations. This study offers a useful perspective by linking cratons, typically regarded as stable and largely unaffected by subduction, to mineralisation along their margins. I recommend acceptance after minor revision.

Main comment:

C1: The authors now make it clear that the numerical models are not intended to directly simulate crustal processes, but rather to identify a first-order geodynamic control (subduction-driven mantle return flow) that may precondition craton margins for mineralisation. This clarification largely resolves my previous concerns about the link between deep lithospheric strain and shallow crustal metallogenesis. However, it should keep a clear distinction between model results in this study and inferred geological processes in the discussion.

R1: Response

We thank the reviewer for this positive assessment. In the revised Discussion, we now maintain a clearer distinction between model-derived results and inferred geological

processes throughout. Specifically, we state that the numerical experiments isolate a first-order lithospheric-scale forcing mechanism rather than directly simulating basin development or ore formation, and we refer to rifting, permeability enhancement, fluid flow, and metallogenesis consistently as interpretive, probabilistic inferences built upon the observed statistical patterns and the geodynamic strain field, rather than as direct outputs of the models.

Minor suggestions:

C2: In Figure 1, the symbol size appears to represent deposit size, but this relationship is not explained in the figure legend. Please clarify this.

R2: Response

Thank you for noting this. We have revised the Figure 1 legend to state explicitly that symbol size scales with deposit size.

C3: In Figure 4, the lithosphere–asthenosphere boundary appears to contain green-coloured regions, whereas this colour does not appear in the corresponding colour bar. Please re-check the plotting routine and colour bar settings.

And all Figure references and captions should be checked for consistency.

R3: Response

Thank you for this careful observation. We have corrected the Figure 4 colour bar and legend so that all plotted colours, including the lithosphere–asthenosphere boundary indicator, are defined clearly. We have also checked all figure references and captions for consistency throughout the manuscript.

Additional feedback:

C4: First, I agree with Reviewer #1 that the proposed mechanism is not directly demonstrated and remains partly inferential. More broadly, the mechanism remains primarily supported by statistical relationships and probabilistic inference, and the process-based constraints remain somewhat uncertain. In addition, the predicted subduction-induced mantle flow likely depends on several other parameters that are not fully explored in the current framework. The treatment of subduction geometry is also quite simplified (e.g. slab angles limited to 5°–30°), whereas natural systems span a much wider range (e.g. Ficini et al., 2017). In addition, possible effects of residual slabs or inherited subduction structures beneath cratons are not considered and could be briefly acknowledged.

Some figures would benefit from closer checking. For example, Fig. 3 could be presented more clearly. There is noticeable overlap between the deposits and the random points, and the overall pattern appears rather scattered, so a more cautious interpretation would be helpful. In Fig. 4, the lithosphere–asthenosphere boundary appears to include green-coloured regions, but this colour is not shown on the corresponding colour bar, making the figure difficult to interpret. In addition, the code shared on Zenodo appears to remain at last year’s version and does not seem to include the ASPECT component. This makes it difficult for reviewers to fully assess and reproduce the modelling results.

The main issue is how the current results are interpreted. The authors should focus more on

explaining why these parameters were chosen and how they relate to the geological conditions of deposit formation, rather than carrying out further modelling.

In my view, the remaining issues mainly concern the level of constraint and the way the results are presented. A clearer distinction between model results and geological interpretation, together with more cautious wording and some additional discussion, should be sufficient to address them.

R4: Response

We thank the reviewer for these thoughtful suggestions. We agree that the principal remaining issue is not the generation of additional model runs, but rather the level of constraint claimed for the existing results and the way those results are interpreted. In response, we have revised the manuscript to separate model results more clearly from geological interpretation, to explain the rationale for the chosen parameter space more directly, and to adopt more cautious wording throughout.

We now state explicitly that the proposed mechanism remains partly inferential and is constrained primarily by the combination of statistical relationships and simplified geodynamic experiments. We also expand the discussion of model limitations to acknowledge that the simulations explore only a restricted subset of parameter combinations, that slab geometries are simplified relative to the full diversity of natural systems, and that residual slabs or inherited subduction structures beneath cratons are not included. These factors are now identified explicitly as important uncertainties and priorities for future work.

With respect to the figures, we have re-examined the panels highlighted by the reviewer. For Figure 3, we have clarified the caption and associated text to acknowledge more clearly the overlap between deposits and random points and to present the pattern in explicitly probabilistic rather than deterministic terms. For Figure 4, we have corrected the colour bar and legend so that all colours used in the figure, including the lithosphere–asthenosphere boundary indicator, are defined consistently.

Finally, we have updated the Zenodo repository to include the latest version of the code together with the ASPECT configuration files used in this study, so that the numerical experiments can be inspected and reproduced in full.
